# Platinum-Induced Toxicity and Zinc-Based Interventions: A Systematic Review of Preclinical Evidence

**DOI:** 10.3390/ijms27219438

**Published:** 2026-10-23

**Authors:** Vasilis Spyridon Tseriotis, Dimitrios Ampazis, Sofia Karachrysafi, Theodora Papamitsou, Dimitrios Kouvelas, Konstantinos Stergiou, Vasileios Fouskas, Pavlos Pavliis, Dimitris Hatzibougias, Sofia Chatzianagnosti, Evangelia Dimitra Dimaki, Ioannis Konstantinidis, Dimitrios Tsiptsios, Konstantinos Lallas, Aleksandar Sič, Vasileios Keramidas, Charalampos Tastanis, Kyriaki Papadopoulou, Angeliki Cheva, Marianthi Arnaoutoglou

**Affiliations:** 1 Department of Epidemiology and Public Health, University College London, 1-19 Torrington Place, London WC1E 7HB, UK; 2 Laboratory of Clinical Pharmacology, Aristotle University Campus, Aristotle University of Thessaloniki, 54124 Thessaloniki, Greece; 3 Department of Neurology, Agios Pavlos General Hospital of Thessaloniki, Leoforos Ethnikis Antistaseos 161, Kalamaria, 55134 Thessaloniki, Greece; 4 Department of Respiratory Medicine, Cavan & Monaghan Hospital, H12Y7W1 Lisdarn, Cavan, Ireland; 5 School of Medicine, RCSI University of Medicine and Health Sciences, 123 St. Stephen’s Green, D02YN77 Dublin, Ireland; 6 School of Physics, Clinical and Optometric Sciences, Clinical Measurement Physiology, Technological University Dublin, Park House Grangegorman, 191 North Circular Road, D07EWV4 Dublin, Ireland; 7 Research Team “Histologistas”, Interinstitutional Postgraduate Program “Health and Environmental Factors”, Department of Medicine, Faculty of Health Sciences, Aristotle University of Thessaloniki, 54124 Thessaloniki, Greece; 8 Laboratory of Histology-Embryology, Department of Medicine, Faculty of Health Sciences, Aristotle University of Thessaloniki, 54124 Thessaloniki, Greece; 9 ENT-Clinic, “George Papanikolaou” General Hospital of Thessaloniki, Leoforos Papanikolaou, Exochi, 57010 Thessaloniki, Greece; 10 Private Pathology Laboratory “Microdiagnostics”, Mitropoleos 88, 54621 Thessaloniki, Greece; 11 Third Department of Neurology, Aristotle University of Thessaloniki, 54124 Thessaloniki, Greece; 12 Department of Medical Oncology, School of Medicine, Aristotle University of Thessaloniki, 54124 Thessaloniki, Greece; 13 Faculty of Medicine, University of Belgrade, 11000 Belgrade, Serbia; 14 Department of Cardiology, Agios Pavlos General Hospital of Thessaloniki, Leoforos Ethnikis Antistaseos 161, Kalamaria, 55134 Thessaloniki, Greece; 15 Department of Hygiene, Social & Preventive Medicine and Medical Statistics, School of Medicine, Faculty of Health Sciences, Aristotle University of Thessaloniki, 54124 Thessaloniki, Greece; 16 1st Neurosurgery Department, AHEPA University Hospital of Thessaloniki, Aristotle University of Thessaloniki, Kiriakidi 1, 54636 Thessaloniki, Greece; 17 Department of Pathology, School of Medicine, Aristotle University of Thessaloniki, 54124 Thessaloniki, Greece; 18 Laboratory of Clinical Neurophysiology, AHEPA University Hospital of Thessaloniki, Aristotle University of Thessaloniki, Kiriakidi 1, 54636 Thessaloniki, Greece

**Keywords:** zinc, platinum, chemotherapy, toxicity, rodents, metallothionein, zinc oxide nanoparticles

## Abstract

Zinc may mitigate platinum-induced toxicity through antioxidant, anti-inflammatory and anti-apoptotic effects. We systematically reviewed preclinical in vivo evidence on zinc-based interventions, focusing on organ protection, mechanisms and antitumour efficacy. PubMed, Scopus, Web of Science and supplementary sources were searched for studies in rats and mice. Risk of bias was assessed using SYRCLE, and the findings were synthesised narratively. Database searches identified 1552 records; 25 studies were included following database and supplementary screening. The risk of bias was frequently unclear for sequence generation, allocation concealment and blinding. Most studies investigated cisplatin; fewer evaluated carboplatin, oxaliplatin or combination chemotherapy. Zinc interventions included salts, dietary supplementation, organic complexes and nanoformulations, predominantly administered before platinum exposure. Protective findings were reported across renal, reproductive, nervous, hepatic, haematopoietic and cardiovascular systems. Proposed mechanisms included metallothionein induction, enhanced antioxidant defences and modulation of inflammation and apoptosis. Heterogeneity precluded conclusions about formulation superiority. Limited tumour-bearing evidence indicated that zinc-induced metallothionein may reduce cisplatin activity, whereas bismuth zinc citrate preserved tumour control within the tested model. Methodological weaknesses, heterogeneity and scarce tumour-bearing evidence limit confidence in efficacy and oncological safety. These findings support further preclinical investigation rather than clinical implementation.

## 1. Introduction

Platinum-based chemotherapeutics such as cisplatin, carboplatin and oxaliplatin remain a cornerstone of curative and palliative regimens for many solid tumours, but their use is limited by dose-dependent nephrotoxicity, neurotoxicity, gonadotoxicity and other off-target organ injuries that may be only partially reversible [1,2]. Oxidative stress, mitochondrial dysfunction, inflammation and dysregulated cell death have emerged as central mechanisms across these toxicities [3].

Zinc is an essential trace element with structural and catalytic roles in hundreds of proteins, including numerous transcription factors and antioxidant enzymes. It exerts antioxidant, anti-inflammatory and anti-apoptotic effects, in part through the induction of metallothioneins (MTs), modulation of redox-sensitive signalling pathways and stabilisation of cellular membranes [4,5]. Experimental work outside the platinum field has long suggested that zinc supplementation can mitigate oxidative tissue damage and modulate immune responses [4,6], and several animal studies indicate that zinc pre-treatment or co-treatment may attenuate cisplatin-induced nephrotoxicity and other organ injuries via MT induction and reactive oxygen species buffering [7,8]. In parallel, newer zinc formulations have been developed to enhance bioavailability or target antioxidant effects, including zinc salts, organic chelates, zinc oxide nanoparticles (ZnO-NPs) and zinc-containing nanocomposites [9,10].

Previous reviews have examined related aspects of platinum toxicity and zinc-based interventions. Reviews of cisplatin-induced nephrotoxicity have summarised mechanisms of renal injury and potential protective compounds [11]. A systematic review of ZnO nanoparticles has described both beneficial and toxic effects across biomedical applications, highlighting the importance of concentration and exposure duration [12]. A chemotherapy-focused review has discussed ZnO nanoparticles as potential modifiers of chemoresistance and chemotherapy-related adverse effects [13]. More recently, a comprehensive review considered zinc alongside other micronutrients for mitigating chemotherapy-induced organ toxicity [14]. These reviews provide important context, including the recognition that zinc-based interventions may themselves cause toxicity, but address different combinations of agents, formulations, experimental settings and clinical evidence.

A focused synthesis is therefore needed to organise the in vivo evidence on zinc-based interventions specifically against platinum-induced toxicity (PIT) across organ systems. The central translational question is whether zinc-based interventions can reduce platinum-induced injury to normal tissues without compromising antitumour efficacy and which experimental conditions support or limit that possibility. The present review addresses this need by systematically evaluating studies in rats and mice encompassing conventional zinc salts, dietary supplementation, organic complexes and nanoformulations. We organise findings by organ/system and chemotherapy regimen, examine formulation and administration timing as potential sources of variation, and integrate evidence concerning MT, oxidative stress, inflammation and apoptosis. We also assess the available tumour-bearing evidence to distinguish organ-protective findings from evidence of preserved antitumour efficacy. This approach aims to clarify the scope and limitations of the preclinical evidence and identify priorities for further investigation, rather than establish comparative superiority or clinical effectiveness.

## 2. Methods

Our study was reported according to the PRISMA statement [15]. The study protocol was registered on PROSPERO (registration number CRD420261349354).

### 2.1. Search Strategy and Information Sources

We searched PubMed, Scopus and Web of Science from inception through 11 June 2026, using free-text terms and controlled vocabulary for zinc, platinum agents and rodent models. The search strategies and numbers of records retrieved from these databases are provided in Supplementary Table S1. We also conducted forward and backward citation searching and supplementary searches in Google Scholar and ProQuest. For each supplementary source, we screened the first 1000 records displayed, which comprised 2000 records across the two sources before deduplication. These searches did not encompass all results returned. The queries, search date, reported result counts and numbers screened are provided in Supplementary Table S2.

### 2.2. Eligibility Criteria

We included experimental studies using rat and mouse models for the in vivo investigation of PIT. These species were selected because they provide established experimental models of platinum-induced organ injury in which systemic drug exposure, tissue pathology, biochemical changes and functional outcomes can be evaluated under controlled treatment conditions [16,17]. Rodent models also permit mechanistic investigation using genetically modified animals, including MT-deficient mice, which is particularly relevant to evaluating MT-dependent effects of zinc interventions [18]. Included studies had to clearly target one or more organs/systems in the context of PIT and include platinum-based compounds as well as zinc supplementation in any form. Regimens of platinum-based chemotherapy and zinc supplementation had to be described in terms of timing, dosage, duration, and potential escalation, as well as assessment of outcomes. In studies evaluating platinum-based compounds in combination with other chemotherapeutics (e.g., BEP regimens), these were considered eligible as long as the platinum agent remained central to the toxicity model; such studies were presented and interpreted separately to account for the added complexity of combination therapy. Eligible studies had to include a toxicity control group receiving the platinum-containing regimen without the zinc intervention. To enable a comprehensive synthesis of mechanistic evidence on zinc supplementation in PIT, we did not restrict eligibility based on predefined outcome measures; instead, we included all otherwise eligible studies regardless of the experimental endpoints or assessment methods employed. Similarly, the inclusion of tumour-bearing models was not mandatory; studies in non-tumour-bearing animals were included, alongside tumour-bearing models where available. Lastly, all eligible studies had to be peer-reviewed and provide their full report in English.

We excluded studies not fulfilling the above-mentioned eligibility criteria. We also excluded studies performed in human subjects or in vitro. Google Scholar and ProQuest were used as supplementary discovery sources to identify full-text articles published in peer-reviewed journals, which were eligible if they met the remaining inclusion criteria; theses, dissertations, conference abstracts and other non-journal reports were excluded.

### 2.3. Data Management

We deduplicated articles on Systematic Review Accelerator [19] and used the online platform Rayyan for article screening [20]. Eligible studies were imported to reference manager “Mendeley” (Desktop version 1.19.8).

### 2.4. Study Selection

**Figure 1:**
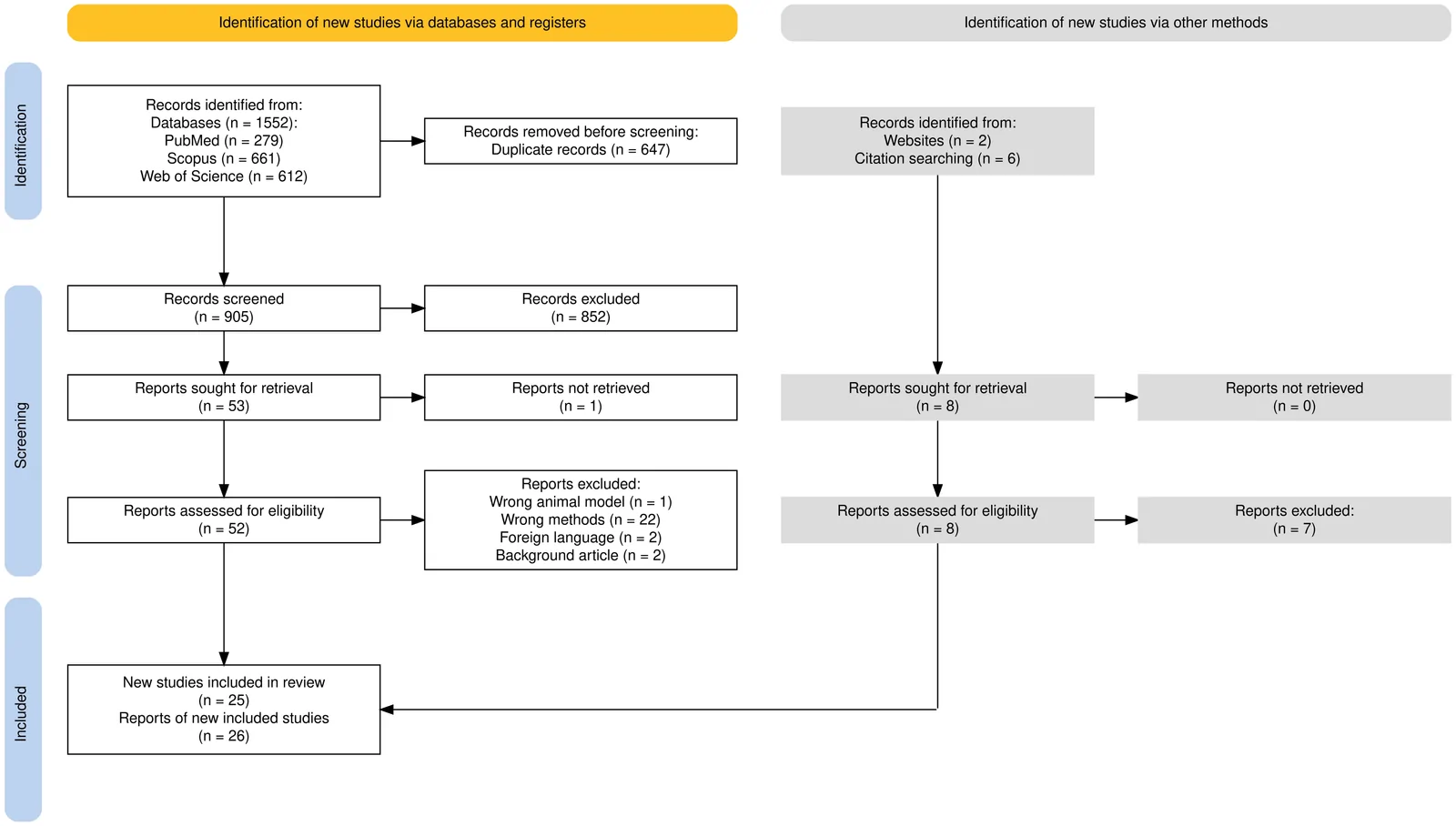
**PRISMA flow diagram.** The two records identified through websites were retrieved following screening of 1000 search results from Google Scholar and 1000 from ProQuest (2000 results in total).

Article screening was conducted in two phases: abstracts and titles and, subsequently, full articles. Screening was performed by two independent reviewers (VST and KL), who were blinded to each other’s decisions. Conflicts were resolved with the involvement of a third investigator (TP). An interactive R-based online tool [21] was used for the construction of the PRISMA flow diagram (Figure 1).

### 2.5. Data Extraction and Quality Assessment

Two independent reviewers extracted the following characteristics (VST, KL): first author/publication year, organ/system studied, animal model of PIT, zinc intervention, experimental groups, and main findings. We used Review Manager (RevMan) 5.4 for quality assessment with SYRCLE risk-of-bias tool for animal studies [22]. Throughout quality assessment, the two reviewers worked independently. Disagreements during data extraction or quality assessment were resolved by a third reviewer (PP). Risk of bias was assessed separately for each SYRCLE domain as low, high or unclear; no overall methodological quality score or classification was assigned.

### 2.6. Qualitative Synthesis

We grouped studies according to chemotherapy regimen used and organ/system investigated, presenting their main results in tabular form. A narrative qualitative synthesis of eligible studies was undertaken because substantial heterogeneity in platinum agents and dosing regimens, zinc formulations and administration schedules, organ systems investigated, and outcome measures precluded meaningful quantitative pooling. Within each organ/system, findings were interpreted in relation to species, platinum agent, zinc formulation, dose and administration timing, together with the domain-specific SYRCLE judgements. Confidence in the interpretation was discussed qualitatively, considering methodological limitations, consistency of findings, differences in experimental models and the availability of functional and antitumour outcomes; no formal certainty-of-evidence grade was assigned. Normal-tissue toxicity and antitumour efficacy were treated as two equally important outcome domains in interpreting translational potential. Available antitumour findings were organised by tumour model, platinum agent, zinc formulation and dose, and timing relative to chemotherapy. Tumour growth or volume, tumour cell survival, animal survival, apoptosis and resistance-related findings were considered where available, distinguishing tumour outcomes from survival under toxic chemotherapy exposure and mechanisms measured in normal tissues.

## 3. Results

### 3.1. Overview of Eligible Studies

Among 1552 search results, 647 were removed after deduplication and 905 were kept. After completion of the abstract and full-text screening, 25 studies investigating Zn supplementation in animal models of PIT were included, published between 1989 and 2024 [8,18,23,24,25,26,27,28,29,30,31,32,33,34,35,36,37,38,39,40,41,42,43,44,45]. Figure 1 presents the PRISMA flow diagram of our work. Most eligible studies tested zinc interventions in experiments with platinum-based chemotherapy alone (Table 1, Supplementary Table S3), whereas, in some studies, additional chemotherapeutics were utilised (Table 2, Supplementary Table S4), e.g., the combination of bleomycin, etoposide, and cisplatin (BEP), mimicking clinically relevant scenarios across multiple organs and rat/mouse strains. Table 1 and Table 2 provide concise summaries of the included studies; detailed animal characteristics, chemotherapy and zinc dosing regimens, administration routes and schedules, experimental groups, sample sizes, and endpoint-specific findings are presented in Supplementary Tables S3 and S4. Among platinum-based chemotherapeutics, cisplatin was the most frequently used agent, administered either as a single bolus or in repeated weekly/cyclic schedules to induce nephrotoxicity, hepatotoxicity, cardiotoxicity, testicular toxicity, and neurotoxicity [8,18,23,24,25,26,27,28,29,30,31,32,33,34,35,37,38,40]. Carboplatin was used to model bone marrow toxicity [41], whereas oxaliplatin primarily induced central and peripheral neurotoxicity [36]. BEP studies addressed testicular and reproductive toxicity [42,43,44,45].

Among the included studies, 16 used rats only, eight used mice only, and one included both species, spanning Sprague-Dawley, Wistar and other laboratory strains of various ages. Three studies included tumour-bearing experiments to evaluate antitumour activity alongside organ protection: intracerebral 9L gliosarcoma in rats, subcutaneous MBT-2 bladder tumours in mice, and orthotopic SKNLP neuroblastoma xenografts in immunodeficient mice [25,30,41].

A broad spectrum of zinc formulations and regimens was used. Classical inorganic salts and dietary zinc included zinc acetate, zinc sulphate, zinc chloride, zinc nitrate, zinc carbonate, and high-zinc diets, as well as zinc gluconate; zinc was also provided as part of multi-antioxidant cocktails with vitamins and selenium [18,23,24,25,26,27,28,29,35,41,42,43,44]. More recent studies used zinc oxide (ZnO)-based systems, including bulk ZnO, conventional ZnO nanoparticles, plant-derived “green” ZnO-NPs, ZnO nanocomposites [such as quaternary ammonium chitosan Schiff base ZnO nanocomposites (QACSB-ZnO)], zinc picolinate, bismuth zinc citrate (BiZn), and ZnO-NPs with different surface chemistries [8,30,32,37,38,39,40]. Zinc was most often given as pre-treatment (±co-treatment) before chemotherapy initiation, but several studies used post-chemotherapy supplementation to support recovery of organ function, particularly in the testis [35,44].

Across studies, two key sources of heterogeneity were consistently identified and are important for interpreting the findings: (i) zinc formulation, ranging from classical salts and dietary zinc to nanoengineered systems such as ZnO nanoparticles, BiZn, and nanocomposites; and (ii) timing of administration, including preventive regimens (pre-treatment ± co-treatment) and post-chemotherapy recovery strategies. These dimensions are considered throughout the following organ-specific analyses, as they appear to influence both the magnitude and the translational relevance of the observed effects.

Detailed results are presented by organ/system, with mechanistic readouts (metallothionein, oxidative stress, inflammation, apoptosis, and impact on antitumour activity) described within each subsection.

### 3.2. Quality Assessment

**Figure 2:**
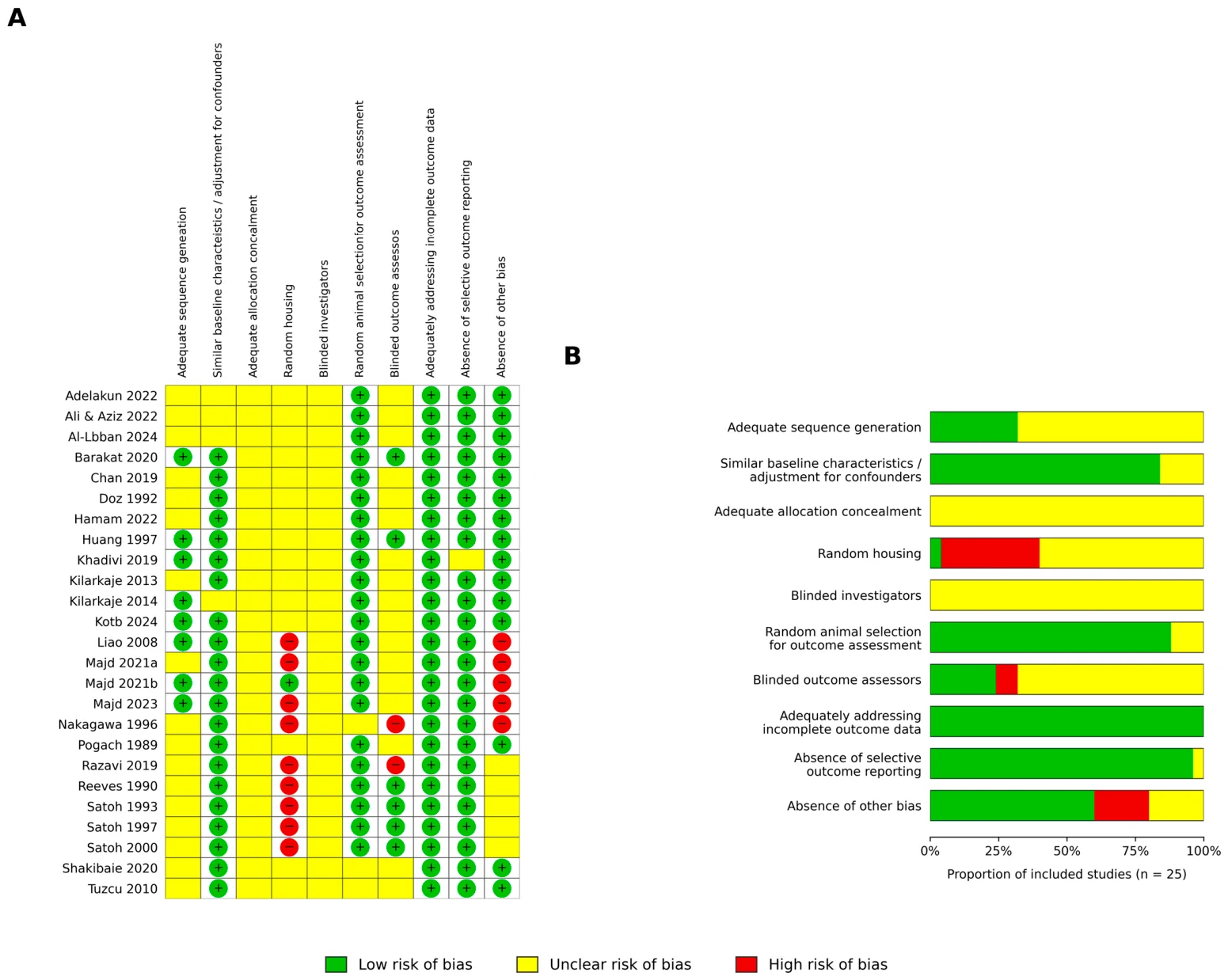
**Risk-of-bias assessment of included animal studies using the SYRCLE risk-of-bias tool.** (**A**) Risk-of-bias summary showing authors’ judgements for each methodological domain across individual studies. Green, red, and yellow cells indicate low, high, and unclear risk of bias, respectively. (**B**) Risk-of-bias graph displaying the proportion of studies rated as low (green), high (red), or unclear risk of bias (yellow) for each domain. Studies shown in panel A are: Adelakun et al., 2022 [35]; Ali and Aziz, 2022 [36]; Al-Lbban, 2024 [39]; Barakat et al., 2020 [31]; Chan et al., 2019 [30]; Doz et al., 1992 [41]; Hamam et al., 2022 [37]; Huang et al., 1997 [27]; Khadivi et al., 2019 [45]; Kilarkaje et al., 2013 [42]; Kilarkaje, 2014 [43]; Kotb et al., 2024 [40]; Liao et al., 2008 [29]; Majd et al., 2021a [33]; Majd et al., 2021b [34]; Majd et al., 2023 [38]; Nakagawa et al., 1996 [26]; Pogach et al., 1989 [23]; Razavi et al., 2019 [44]; Reeves et al., 1990 [24]; Satoh et al., 1993 [25]; Satoh et al., 1997 [18]; Satoh et al., 2000 [28]; Shakibaie et al., 2020 [32]; and Tuzcu et al., 2010 [8].

The SYRCLE assessment identified domain-specific risk-of-bias concerns, particularly unclear risk for sequence generation, allocation concealment and blinding and high risk for random housing in several studies (Figure 2). Most studies were judged as low risk for baseline comparability, incomplete outcome data, and selective outcome reporting, which indicates adequate group matching, data completeness, and consistency between reported outcomes and study aims. Low risk was also commonly observed for random outcome assessment and absence of other bias.

By contrast, critical methodological domains related to internal validity were frequently underreported. Sequence generation, allocation concealment, and blinding of investigators and outcome assessors were seldom described, which predominantly resulted in an unclear risk of bias in these domains. Random housing was either not reported or explicitly non-random in several studies, which led to a notable proportion being rated as high risk for this domain. Collectively, these limitations suggest that while outcome integrity was generally adequate, insufficient reporting of randomisation and blinding procedures reduces confidence in the magnitude and consistency of the reported protective effects, may permit the overestimation of benefits, and should be considered when interpreting the findings.

### 3.3. Organ- and System-Specific Effects of Zinc

#### 3.3.1. Nephrotoxicity

Across cisplatin models, nephrotoxicity was consistently characterised by elevated BUN/creatinine, tubular necrosis, and increased lipid peroxidation with reduced antioxidant defences [8,18,24,25,26,27,30,31,38].

Mechanistic studies showed that zinc frequently induced renal metallothionein (MT) but its protective impact depended on context. Dietary zinc increased baseline kidney and liver MT, and cisplatin further increased kidney MT, yet high-zinc diets did not reduce cisplatin-induced rises in serum urea or weight loss, likely due to concurrent copper deficiency [24]. By contrast, short zinc pre-treatment with ZnSO_4_ or ZnCl_2_ in mice strongly induced renal MT and prevented cisplatin-related increases in blood urea nitrogen (BUN) and lipid peroxidation, including under pharmacological glutathione depletion, with clear histological protection in wild-type but not MT-null animals [18,25,26,28]. In these knockout models, zinc failed to confer nephroprotection, which indicates that renal protection requires MT induction [18,28].

Several studies evaluated functional nephroprotection with zinc salts and small molecules. Zinc gluconate reduced cisplatin-induced elevations in serum creatinine, BUN, and renal malondialdehyde (MDA) while improving glutathione status, although its effects on individual renal antioxidant enzymes were modest [27]. Zinc picolinate after cisplatin significantly improved BUN and creatinine, reduced renal oxidative and inflammatory markers, reduced Bax expression while Bcl-2 expression remained unchanged, and decreased stress protein expression, with improved tubular histology [8].

More recent ZnO-based formulations showed substantial protective signals, often with dose-dependent patterns. ZnO-NPs given prior to cisplatin largely normalised kidney function markers, improved superoxide dismutase (SOD)/catalase (CAT)/glutathione (GSH), decreased MDA, reduced apoptosis and transforming growth factor-β1 (TGF-β1) expression, restored Nrf2/HO-1/eNOS pathways, and enhanced tubular regeneration [31]. In a longer cisplatin protocol, bulk ZnO, chemical ZnO-NPs, and “green” ZnO-NPs all reduced BUN, lipid peroxidation, and tubular/glomerular damage, with green ZnO-NPs showing more pronounced protective findings within that study across oxidative stress, apoptosis markers, and histology [38]. BiZn given before and during cisplatin treatment similarly improved BUN, creatinine, tubular injury scores, and survival at lethal cisplatin doses, in association with increased renal MT and GSH; complementary experiments in cultured HK-2 renal cells showed that MT-IIA knockdown or inhibition of GSH synthesis reduced BiZn-associated protection, supporting the involvement of these systems in the cellular model [30].

Overall, the renal evidence comprised ten cisplatin studies, including four rat and six mouse studies. Zinc interventions included dietary zinc at 5–320 mg Zn/kg, subcutaneous zinc sulphate or chloride at 100–200 µmol/kg, oral zinc gluconate at 100–180 mg/kg/day, intraperitoneal zinc picolinate at 6 mg elemental Zn/kg/day, ZnO-based formulations at 5 mg/kg, and oral BiZn at 0.14 mmol/kg. Regimens predominantly involved pre-treatment, sometimes continued during or after cisplatin exposure (Supplementary Table S3). Protection varied across these conditions: MT-dependent effects were supported by knockout experiments, whereas increased MT following dietary supplementation did not consistently translate into reduced renal injury. Allocation concealment and investigator blinding were unclear across these studies, and several received high-risk judgements for random housing (Figure 2). These limitations reduce confidence in the magnitude of nephroprotection; differences in species, doses, formulations and chemotherapy schedules also prevent reliable comparisons between interventions.

#### 3.3.2. Reproductive and Gonadal Toxicity

Both cisplatin-only and BEP-based regimens produced marked testicular and reproductive toxicity, including impaired spermatogenesis, altered sperm quality, and endocrine disturbances [23,32,33,35,37,42,43,44,45]. Cisplatin reduced LH, FSH, serum and testicular testosterone and androgen-binding protein (ABP) and disrupted spermatogenic indices and seminiferous tubule architecture [23,35]. BEP regimens similarly decreased sperm count and motility, increased abnormal morphology, caused tubular atrophy, and impaired hormonal profiles and fertility [42,43,44,45].

Zinc’s reproductive effects varied by formulation and timing. Zinc acetate given as a short pre-treatment and co-treatment did not prevent cisplatin-induced suppression of luteinising hormone/follicle-stimulating hormone (LH/FSH) or testosterone, and zinc acetate alone also reduced testicular testosterone; however, it partially preserved Sertoli cell function, as reflected by near-normal ABP levels, which indicates selective support of Sertoli function but not Leydig or pituitary recovery [23]. By contrast, prolonged oral zinc sulphate given for an entire spermatogenic cycle after a single cisplatin dose substantially restored sperm count, motility, and morphology; daily sperm production; germ cell numbers; testicular morphometry; and gonadotropin/testosterone levels, together with improved antioxidant status and seminiferous epithelium integrity [35].

Several studies suggested that ZnO and ZnO-NPs may provide greater gonadal protection than simple salts. ZnO-NPs, especially at higher doses, reduced testicular lipid peroxidation, restored glutathione and antioxidant enzymes, and preserved seminiferous architecture and spermatogenesis more effectively than ZnSO_4_, approaching the protection achieved by vitamin E comparators [32]. In adult rats, “green” ZnO-NPs were associated with the most complete protection within that study against cisplatin-induced reductions in testis weight, seminiferous integrity, sperm parameters, oxidative stress, testosterone levels, and steroidogenic gene expression, outperforming both bulk ZnO and conventional ZnO-NPs [33]. In prepubertal models, ZnO-NP/cisplatin co-administration preserved blood–testis barrier markers, reduced oxidative and inflammatory changes, improved sperm quality, and restored chromatin/protamine integrity over time [37].

In BEP models, antioxidant cocktails containing zinc modestly attenuated the acute impact of BEP on sperm parameters and partially supported the recovery of spermatogenesis, germ cell repopulation, and endocrine function over a 63-day post-treatment period but did not prevent significant initial testicular damage [42,43]. Post-BEP zinc sulphate alone, given for one full spermatogenic cycle after chemotherapy, improved sperm concentration, motility, and morphology; chromatin condensation; and seminiferous architecture and restored spermatogonia and Leydig cell counts, although some parameters remained below control levels [44]. A BEP model focusing on epigenetic and chromatin endpoints showed that post-BEP zinc supplementation improved DNA methylation and protamination, reduced DNA fragmentation and ubiquitination, and supported testicular structural recovery, again indicating an incomplete but biologically meaningful recovery of reproductive function [45].

Overall, reproductive outcomes were investigated in nine studies, which comprised eight rat studies and one mouse study; five evaluated cisplatin alone, and four evaluated BEP. Interventions included intraperitoneal zinc acetate at 6 mg/kg/day, oral zinc sulphate at 1 mg/kg/day after cisplatin or 10 mg/kg/day after BEP, zinc nanoparticles at 10–50 mg/kg orally, ZnO formulations at 5 mg/kg intraperitoneally, and orally administered antioxidant cocktails containing zinc at 40 mg/L. Schedules ranged from pre-treatment and co-treatment to prolonged post-chemotherapy supplementation (Supplementary Tables S3 and S4). The findings indicate selective protection or partial recovery, with differences in developmental stage, chemotherapy exposure and supplementation duration limiting comparisons. Allocation concealment and investigator blinding were unclear throughout this evidence group, while outcome-assessor blinding was predominantly unclear and some studies received high-risk judgements for housing or assessor blinding (Figure 2). These concerns limit confidence in the magnitude and consistency of the benefit. Effects observed with antioxidant cocktails cannot be attributed to zinc independently of their other components.

#### 3.3.3. Neurotoxicity

In both the central and peripheral nervous system (CNS and PNS, respectively), cisplatin and oxaliplatin induced oxidative stress, neurotrophin depletion, and structural brain injury, accompanied by behavioural abnormalities [34,36]. In hippocampal models, cisplatin decreased antioxidant enzymes, shifted glutathione redox balance towards oxidation, increased lipid peroxidation, reduced NGF and BDNF expression, and caused neuronal loss and thinning of pyramidal and granular layers in CA1, CA3, and dentate gyrus [34]. Oxaliplatin induced thermal hyperalgesia and cold allodynia, reduced antioxidant enzyme activity, increased glial fibrillary acidic protein (GFAP) and inflammatory markers [nuclear factor-kappa B (NF-κB), tumour necrosis factor-alpha (TNF-α)], and produced cerebral and cerebellar lesions with increased p53 and reduced Bcl-2 [36].

Across both models, zinc-based interventions were associated with improvements in several CNS outcomes. In cisplatin-treated rats, green ZnO-NPs nearly normalised antioxidant enzymes, restored the GSH/GSSG ratio, reduced MDA, recovered NGF and BDNF mRNA, and improved neuronal morphology and cell counts in hippocampal subfields, whereas chemical ZnO-NPs and bulk ZnO achieved only partial biochemical and histological recovery [34]. In the oxaliplatin model, zinc alone partially improved nociceptive responses, modestly restored antioxidant activity, reduced GFAP, and provided limited histological protection; melatonin alone had similar partial benefits [36]. However, zinc–melatonin combination, particularly when given as pre-treatment, was associated with the greatest protection within that model, normalising thermal nociception, enhancing antioxidant defences, markedly suppressing GFAP and p53, and reducing lesion scores more effectively than either agent alone [36].

The neurotoxicity evidence comprised two rat studies with distinct platinum agents and endpoints. The cisplatin study evaluated bulk, chemical and green ZnO formulations at 5 mg/kg/day intraperitoneally for 28 days during repeated chemotherapy exposure, principally assessing hippocampal biochemical and histological outcomes. The oxaliplatin study evaluated oral zinc at 15 mg/kg/day, alone or with melatonin, using pre-treatment followed by co-treatment or co-treatment alone, and it included nociceptive and brain tissue outcomes (Supplementary Table S3). Allocation concealment and investigator and outcome-assessor blinding were unclear in both studies; the cisplatin study also received a high-risk judgement for other bias (Figure 2). Together with the absence of independent replication of either intervention, these limitations constrain confidence in generalising the findings across central and peripheral neurotoxicity. The benefits of zinc–melatonin combinations cannot be attributed to zinc alone.

#### 3.3.4. Hepatotoxicity

Cisplatin caused consistent hepatotoxic changes, including elevated serum transaminases and alkaline phosphatase, reduced hepatic GSH, increased MDA, altered liver weight indices, and histological evidence of hepatocellular degeneration and inflammation [29,40].

In a multifactorial antioxidant design, zinc contributed to partial hepatoprotection [29]. Zinc-containing regimens reduced hepatic lipid peroxidation and improved ALT levels, but no single agent alone completely normalised all liver injury markers. Fosfomycin and methionine appeared to have the most prominent individual effects on liver function and GSH levels, with zinc and selenium mainly reducing oxidative stress and transaminases within combinations. By contrast, a ZnO nanocomposite (QACSB-ZnO) given around cisplatin treatment was associated with the attenuation of hepatotoxicity, improving liver enzymes, serum proteins, hepatic GSH and MDA and reducing TNF-α, with a preserved hepatic architecture and favourable modulation of TGF-β1 and Bcl-2 expression on immunohistochemistry [40].

The hepatic evidence comprised two cisplatin studies: a mouse study evaluating oral zinc sulphate at 25 mg/kg/day within a nine-day multifactor intervention protocol and a rat study evaluating a QACSB–ZnO nanocomposite at 150 mg/kg/day intraperitoneally for 14 days. Both included supplementation before and during chemotherapy (Supplementary Table S3). Allocation concealment and investigator and outcome-assessor blinding were unclear in both studies, while the multifactor study received high-risk judgements for random housing and other bias (Figure 2). Confidence in a reproducible zinc-specific effect is therefore limited by methodological concerns and substantially different intervention designs. Combination effects cannot be assigned to zinc alone, and the nanocomposite study does not isolate the contribution of its zinc component.

#### 3.3.5. Bone Marrow and Haematological Toxicity

Carboplatin significantly reduced colony forming unit–granulocyte–macrophage (CFU-GM) colony formation, which indicates haematopoietic toxicity [41]. A high-zinc diet did not alter baseline CFU-GM counts but was associated with reduced carboplatin-induced myelotoxicity, with higher CFU-GM survival and the ability to escalate carboplatin doses by approximately 50% without additional marrow suppression, coincident with increased MT mRNA in bone marrow and kidney [41].

The marrow evidence was limited to one mouse study using carboplatin and a diet containing 180 ppm zinc, compared with 10 ppm in the control diet, for 12–14 days before chemotherapy (Supplementary Table S3). Sequence generation, allocation concealment, random housing, and investigator and outcome-assessor blinding were unclear (Figure 2). The reported improvement in CFU-GM survival therefore supports a preliminary signal of increased marrow tolerance within this model. However, methodological uncertainty, reliance on a single study and the colony formation endpoint limit confidence in broader or sustained haematological protection.

#### 3.3.6. Cardiovascular Toxicity

Cisplatin induced cardiometabolic toxicity, including dyslipidaemia, unfavourable atherogenic indices, impaired kidney function, cardiac oxidative stress, and elevated inflammatory and cardiac injury markers [39]. Oral ZnO-NPs administered after cisplatin were associated with dose-dependent cardioprotective effects, progressively improving lipid profiles and atherogenic indices, improving renal function markers, enhancing cardiac antioxidant defences, and reducing inflammatory and injury markers, with near-complete normalisation at the highest zinc nanoparticle dose [39].

The cardiovascular evidence was limited to one rat study using a single intraperitoneal cisplatin dose of 20 mg/kg followed by oral ZnO nanoparticles at 10–50 mg/kg/day for 15 days (Supplementary Table S3). Sequence generation, baseline comparability, allocation concealment, random housing, and investigator and outcome-assessor blinding were unclear (Figure 2). Although dose-dependent improvements were reported, these methodological uncertainties and the reliance on predominantly biochemical outcomes reduce confidence in sustained cardiac functional benefit. The findings also cannot establish protection during repeated chemotherapy cycles or the relative effectiveness of other zinc formulations.

### 3.4. Zinc and Antitumour Efficacy of Platinum Agents

Whether normal tissue protection is compatible with maintained antitumour activity is central to the translational interpretation of zinc supplementation. Three included studies provided relevant evidence across distinct tumour models, zinc interventions and platinum agents (Table 3). Their results were therefore considered separately rather than combined into a general conclusion about oncological safety.

With zinc pre-treatment, findings differed between models. In C3H mice bearing MBT-2 bladder tumours, zinc sulphate increased tumour and renal MT, attenuating nephrotoxicity but reducing cisplatin antitumour activity. Propargylglycine inhibited tumour MT induction while retaining renal MT induction, restoring tumour responsiveness without abolishing renal protection [25]. By contrast, Doz et al. evaluated a high-zinc diet before carboplatin exposure and found no reduction in its activity against 9L gliosarcoma in rats, assessed through the colony forming efficiency of tumour-derived cells. MT mRNA induction occurred in extracerebral tissues but not in the 9L tumours, which suggests that tissue-specific induction may influence the balance between protection and tumour response [41].

With pre-treatment followed by continued supplementation, oral BiZn was evaluated in an orthotopic SKNLP neuroblastoma xenograft model. No reduction in cisplatin-mediated tumour control was detected in tumour growth and bioluminescence assessments, including metastatic-site assessments [30]. The survival benefit reported under lethal cisplatin exposure should be interpreted as a toxicity-related finding rather than evidence of improved cancer-specific survival. Similarly, apoptosis findings in renal cells cannot establish the preservation of tumour cell apoptosis.

These studies do not establish equivalence between platinum treatment alone and zinc-containing regimens, nor can they identify an optimal supplementation schedule. None of these tumour experiments evaluated an exclusively post-platinum zinc strategy. Tumour-specific survival, apoptosis and acquired resistance remain insufficiently characterised to support broad conclusions about maintained anticancer efficacy. Allocation concealment and investigator blinding were unclear in the relevant studies (Figure 2), which further limited confidence. Preservation of the tumour response in selected models therefore cannot be generalised across zinc formulations, tumour types or platinum agents.

## 4. Discussion

We herein systematically review in vivo rat/mouse studies evaluating zinc-based interventions against PIT across renal, gonadal, neural, hepatic, haematopoietic and cardiovascular systems. Despite heterogeneity in models and interventions, the included studies generally suggest that zinc may mitigate markers of toxicity across multiple organs. MT responsiveness may help explain the variation in the observed protective effects, although the heterogeneous studies do not establish which tissues benefit most or a common mechanism across all zinc interventions. Limited tumour-bearing data further indicate that zinc’s impact on chemotherapy efficacy is context-dependent and may be influenced by tumour MT inducibility. Because only a small number of studies evaluated tumour-bearing models, any apparent organ-protective benefit must be interpreted cautiously, as it does not necessarily imply preserved antitumour efficacy or clinical safety [25,30,41].

Zinc formulation may influence the observed protective effects and their potential translational relevance. Classical zinc salts and high-zinc diets reliably induced metallothionein and improved oxidative-stress markers but often provided only partial functional protection, and, in some settings, no clear organ protection was observed [23,24,27,29,35,41]. Studies evaluating ZnO nanoparticles, BiZn and engineered nanocomposites reported protective effects across multiple biological endpoints, including oxidative stress reduction, anti-inflammatory signalling and the modulation of apoptosis [8,30,31,32,34,37,38,39]. This is in line with the broader nanomedicine literature and with a previous systematic review showing that ZnO-NPs can enhance chemotherapy delivery and modulate the tumour microenvironment [46]. Apparent differences between nanoformulations and conventional zinc salts should be interpreted cautiously. Nominal doses are not necessarily comparable because studies report amounts of zinc salts, elemental zinc, nanoparticles or composite materials; these do not imply equivalent zinc exposure. Differences in administration route, dissolution, absorption and tissue distribution may also influence exposure, but comparative bioavailability was not established across the included studies. Platinum dosing, supplementation timing, animal characteristics and outcome selection further complicate comparisons. Consequently, broader protective findings in some nanoformulation studies cannot establish an intrinsic formulation advantage. Direct comparisons using characterised formulations and matched elemental zinc doses, together with measurements of systemic and tissue exposure, are needed to assess comparative efficacy.

The timing of administration may influence the observed effects of zinc supplementation. Preventive strategies (zinc pre-treatment with or without co-treatment) were associated with the attenuation of acute toxicity and modulation of oxidative and inflammatory pathways. These approaches are mechanistically informative, but their applicability to clinical chemotherapy schedules remains uncertain. Post-chemotherapy supplementation was generally associated with partial recovery of tissue structure and function, as illustrated in reproductive models where spermatogenesis and chromatin integrity improved but did not fully normalise [35,42,43,44,45]. Peri-cycle or recovery-phase supplementation may be more feasible clinically, although the heterogeneous evidence does not establish the relative effectiveness of different administration schedules.

The limitations of the eligible studies reduce confidence in the reported benefits. Insufficient reporting of sequence generation, allocation concealment and blinding frequently resulted in unclear risk-of-bias judgements, while non-random housing contributed to high-risk judgements in several studies. These concerns, together with small sample sizes and short experimental durations, increase vulnerability to bias and imprecision and warrant cautious interpretation of the magnitude and consistency of the reported protective effects. Acute, high-dose platinum models may have limited clinical relevance compared with clinically realistic multi-cycle regimens, particularly when studies assess short-term biochemical endpoints without parallel functional outcomes [18,24,27,39]. Biological variation may also contribute to differences between studies. Animal age and sex may influence susceptibility to platinum-induced injury and endogenous protective responses, which makes results from prepubertal, adult and aged animals, or from single-sex experiments, potentially non-equivalent [47]. Baseline zinc nutritional status may affect MT inducibility and the response to supplementation; correction of zinc deficiency should therefore be distinguished from additional zinc exposure in zinc-replete animals. In one included dietary study, increased zinc intake induced renal MT without preventing cisplatin toxicity and was accompanied by evidence of mild copper deficiency [24], which illustrates that zinc exposure cannot be interpreted independently of trace element balance. The included evidence does not establish the independent effects of age, sex or baseline zinc status on treatment benefit, so these factors remain potential sources of heterogeneity rather than demonstrated explanations for differences between interventions. For nanotechnology-based interventions, incomplete nanoparticle characterisation, the absence of biodistribution data and limited safety profiling hinder translational interpretation, while dose–response relationships and oral administration routes remain underexplored [32,34,38]. Substantial heterogeneity in platinum agents, dosing schedules, zinc formulations, organ systems studied and outcome measures complicates synthesis and precludes quantitative aggregation, requiring cautious, narrative interpretation of the evidence. An additional major limitation is that only a very small subset of included studies used tumour-bearing models. As a result, the review can more confidently comment on organ-protective signals than on whether those effects are compatible with maintained anticancer efficacy. Finally, the limitations of our review include the restriction to rat/mouse models and English-language reports. Accordingly, our conclusions should be interpreted as applying to a selective preclinical evidence base rather than to the entirety of the experimental evidence. Additional preclinical studies with standardised and clinically aligned protocols are needed before zinc-based strategies can be rationally translated into supportive oncology care.

### 4.1. Mechanistic Synthesis

Across the included studies, the zinc-associated protection of normal tissues involved overlapping changes in MT expression, antioxidant defences, inflammatory signalling and apoptosis. These findings support a mechanistic framework rather than a single established pathway shared by all formulations and organs. Most studies measured biochemical or expression changes alongside tissue injury; such associations do not establish that each pathway was necessary for protection.

The most direct in vivo evidence concerns MT. Comparisons between wild-type and MT-null mice support an MT-dependent component of zinc-mediated renal protection [18,28]. In GSH-depleted mice, renal protection following zinc-induced MT was attributed principally to antioxidant activity rather than increased platinum binding [26]. MT induction and redox buffering therefore provide a better-supported explanation than direct platinum sequestration. Complementary experiments within the BiZn study implicated MT and GSH in the protection of cultured renal cells, but these mechanistic interventions should be distinguished from its animal experiments [30].

A second component involves oxidative stress and its relationship with inflammation and apoptosis. Zinc-containing interventions were associated with reduced lipid peroxidation and the preservation or restoration of GSH and antioxidant enzyme activities across several organs [8,27,31,32,34,38,39,40]. Increased renal Nrf2 and HO-1 expression was reported with ZnO nanoparticles, supporting the involvement of antioxidant response signalling in that model [31]. Reduced NF-κB-related inflammatory markers and changes in TNF-α, IL-6, Bax, p53 and Bcl-2 were also reported in selected models [8,31,36,37,38,40]. These observations suggest interconnected responses to tissue injury but do not establish Nrf2 activation, NF-κB suppression or inhibition of apoptosis as universal mechanisms of zinc-mediated protection.

Importantly, these responses cannot be assumed to be selective for normal tissues. The MBT-2 experiment provides direct evidence that zinc-associated tumour MT induction can accompany reduced cisplatin activity, whereas the 9L experiment showed no detectable loss of carboplatin activity when tumour MT mRNA was not induced [25,41]. BiZn preserved measured cisplatin tumour-control outcomes in the SKNLP model but did not establish that every protective pathway was restricted to normal cells [30]. Whether enhanced antioxidant responses or the suppression of apoptosis also support tumour cell survival remains a mechanistic concern requiring direct testing. Likewise, reduced inflammatory signalling in normal organs does not establish a favourable effect on tumour response. The relevant translational distinction is therefore between the demonstrated protection of normal tissues and the demonstrated preservation of platinum sensitivity in tumours.

**Figure 3:**
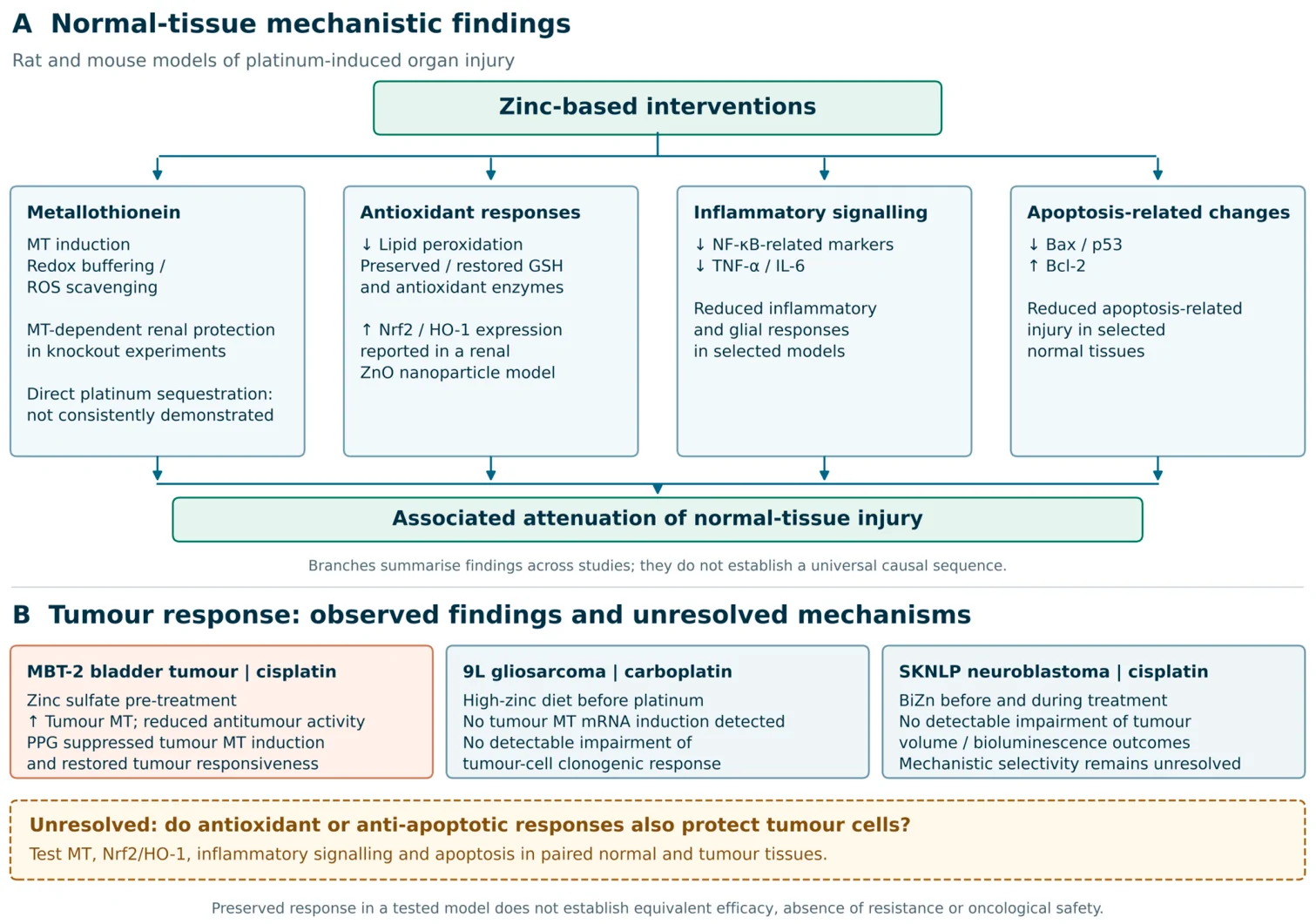
**Mechanistic synthesis of zinc-associated normal tissue protection and tumour response findings in platinum-treated rat and mouse models**. (**A**) Reported changes in metallothionein, antioxidant responses, inflammatory signalling and apoptosis associated with attenuation of normal-tissue injury. Branches integrate findings across different studies and do not indicate that every pathway was demonstrated in every model or establish a universal causal sequence. (**B**) Tumour response findings in the MBT-2, 9L and SKNLP experiments. The dashed box identifies unresolved questions concerning protection of tumour cells. Preserved tumour response in selected experiments does not establish equivalent antitumour efficacy or oncological safety. BiZn, bismuth zinc citrate; GSH, reduced glutathione; HO-1, heme oxygenase-1; IL-6, interleukin-6; MT, metallothionein; NF-κB, nuclear factor-κB; Nrf2, nuclear factor erythroid 2-related factor 2; PPG, propargylglycine; ROS, reactive oxygen species; TNF-α, tumour necrosis factor-α; ZnO, zinc oxide.

Figure 3 integrates the reported normal tissue mechanisms with the available tumour response findings and highlights unresolved questions concerning mechanistic selectivity.

### 4.2. Evidence Gaps and Research Priorities

The first priority is to evaluate normal tissue protection and antitumour efficacy within the same tumour-bearing experiments. Studies should combine organ-specific functional and histological outcomes with tumour growth, tumour cell apoptosis and survival, distinguishing treatment-related mortality from cancer progression. Repeated treatment and longer follow-up are needed to assess sustained tumour control, delayed toxicity and the development of resistance.

The second priority is to establish whether protective mechanisms differ between normal and tumour tissues. Paired measurements of MT, Nrf2/HO-1, NF-κB-related signalling, redox status and apoptosis should be combined, where feasible, with genetic or pharmacological perturbation to test causal involvement. Tissue platinum exposure and platinum–DNA adduct measurements would help distinguish changes in drug delivery from changes in cellular susceptibility. These experiments should also quantify zinc accumulation over time in plasma, vulnerable normal organs and tumour tissue, accounting for baseline zinc status, formulation, dose and administration route. Tissue zinc measurements should be interpreted alongside local MT induction, redox responses and platinum sensitivity rather than treating accumulation alone as evidence of selective protection. Preferential exposure of normal tissues could support organ protection, whereas tumour zinc accumulation accompanied by cytoprotective responses could compromise platinum activity; these possibilities require direct evaluation within the same experimental model. Such experiments should determine whether organ protection can be achieved without activating protective responses in the tumour.

The third priority is to define reproducible intervention conditions. Comparisons should address zinc formulation, elemental zinc exposure, route and timing before, during and after clinically relevant platinum schedules. Baseline zinc status, zinc-related toxicity and, for nanoformulations, particle characterisation and biodistribution should be assessed. Reproducibility should be assessed across age groups, sexes, rat and mouse models, and tumour types, with baseline dietary zinc intake and zinc status documented before supplementation. Adequately justified sample sizes, randomisation, allocation concealment and blinded outcome assessment are needed to strengthen confidence. Until these gaps are addressed, the evidence supports further preclinical investigation rather than selection of a preferred formulation or administration schedule for clinical use.

**Figure 4:**
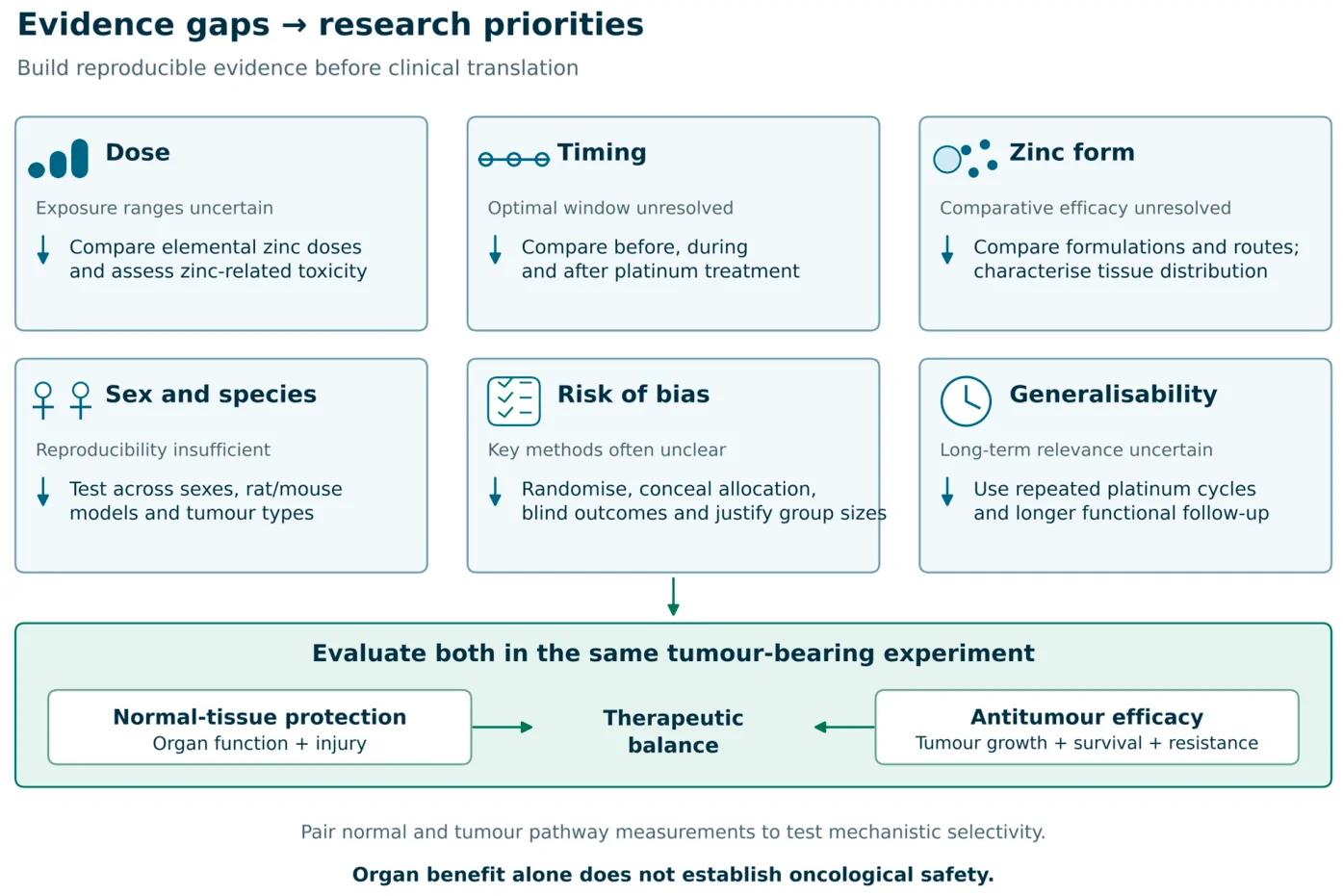
**Evidence gaps and future research roadmap for zinc-based interventions against platinum-induced toxicity.** The roadmap links limitations identified in the narrative synthesis to research priorities concerning paired normal tissue and tumour outcomes, mechanistic selectivity, zinc dose and baseline status, administration timing, formulation and route, sex and species, risk of bias, and generalisability. The central translational question is whether reproducible organ protection can be achieved while maintaining antitumour efficacy and acceptable zinc safety. These priorities do not constitute established treatment recommendations or a formal certainty-of-evidence assessment.

Figure 4 summarises the principal evidence gaps and corresponding priorities for future preclinical research.

## 5. Conclusions

Zinc-based interventions were associated with the attenuation of platinum-induced injury in several rodent organ systems. MT induction, changes in antioxidant defences, inflammatory signalling and apoptosis provide plausible explanations, although causal support varies across pathways and experimental models. Heterogeneity and domain-specific risk-of-bias concerns preclude firm conclusions about comparative efficacy or optimal administration schedules. Evidence of normal tissue protection is more extensive than evidence of preserved antitumour efficacy, and tumour MT induction was associated with reduced cisplatin activity in one included model. These findings support further preclinical investigation but do not establish oncological safety or justify clinical implementation. Studies that evaluate organ protection and tumour response together are required to determine whether zinc-based interventions can improve the therapeutic balance of platinum chemotherapy.

## Figures and Tables

**Table 1:** Summary of animal studies investigating zinc-based interventions against cisplatin-, carboplatin- or oxaliplatin-induced toxicity.

Study	Organ/System	Animal Model and Platinum Agent	Zinc Formulation and Timing	Key Findings
Pogach et al., 1989 [23]	Reproductive	Rats; cisplatin	Zinc acetate; pre-treatment and co-treatment	Partially preserved Sertoli cell androgen-binding protein but did not prevent hormonal disturbances or Leydig/pituitary dysfunction.
Reeves et al., 1990 [24]	Kidney	Rats; cisplatin	Dietary zinc carbonate; pre-treatment and continued supplementation	Increased metallothionein but did not reduce nephrotoxicity. High zinc intake was associated with reduced copper status.
Doz et al., 1992 [41]	Bone marrow; tumour response	Mice and rats; carboplatin	High-zinc diet; pre-treatment	Reduced myelotoxicity and increased metallothionein expression; permitted higher carboplatin doses without additional marrow toxicity in the tested model. No reduction in carboplatin activity against intracerebral 9L gliosarcoma was detected in the rat tumour experiment.
Satoh et al., 1993 [25]	Kidney; tumour response	Tumour-bearing mice; cisplatin	Zinc sulphate; pre-treatment	Reduced nephrotoxicity but also reduced antitumour activity. Inhibition of tumour metallothionein induction restored tumour response while retaining renal protection.
Nakagawa et al., 1996 [26]	Kidney	Mice; cisplatin with glutathione depletion	Zinc chloride; pre-treatment	Attenuated glutathione-depletion-enhanced renal injury. Findings supported metallothionein-associated redox protection without increased platinum binding to metallothionein.
Huang et al., 1997 [27]	Kidney	Mice; cisplatin	Zinc gluconate; pre-treatment and co-treatment	Improved renal-function markers and reduced lipid peroxidation, with limited effects on some antioxidant enzymes.
Satoh et al., 1997 [18]	Kidney	Wild-type and metallothionein-null mice; cisplatin	Zinc sulphate; pre-treatment	Protected wild-type mice but not metallothionein-null mice, supporting a metallothionein-dependent protective effect.
Satoh et al., 2000 [28]	Kidney	Wild-type and metallothionein-null mice; cisplatin with or without glutathione depletion	Zinc sulphate; pre-treatment	Reduced renal injury in wild-type mice, including under glutathione depletion; protection was absent in metallothionein-null mice.
Liao et al., 2008 [29]	Liver	Mice; cisplatin	Zinc sulphate within a multifactor intervention design; supplementation around chemotherapy	Zinc-containing interventions improved selected hepatic injury and oxidative-stress markers; findings arose from a design evaluating several protective agents.
Tuzcu et al., 2010 [8]	Kidney	Rats; cisplatin	Zinc picolinate; supplementation before and after cisplatin	Improved renal function and histological/mechanistic markers of oxidative stress, inflammation and apoptosis; did not prevent weight loss.
Chan et al., 2019 [30]	Kidney; survival; tumour response	Mice, including a neuroblastoma xenograft model; cisplatin	Bismuth zinc citrate; pre-treatment and continued supplementation	Reduced renal injury and improved survival under lethal dosing. No reduction in cisplatin tumour control was detected in the tested xenograft model.
Barakat et al., 2020 [31]	Kidney	Rats; cisplatin	ZnO nanoparticles; pre-treatment	Improved renal-function markers, antioxidant status and histopathology, with changes in inflammatory, apoptotic and regenerative markers.
Shakibaie et al., 2020 [32]	Reproductive	Mice; cisplatin	Plant-mediated zinc nanoparticles or zinc sulphate; pre-treatment and co-treatment	Improved testicular oxidative-stress markers and histology; responses varied by nanoparticle dose and zinc formulation.
Majd et al., 2021a [33]	Reproductive	Rats; repeated cisplatin	Bulk, chemically synthesised or green ZnO nanoparticles; co-treatment	Improved testicular, sperm and endocrine outcomes, with formulation-dependent responses within the study.
Majd et al., 2021b [34]	Central nervous system	Rats; repeated cisplatin	Bulk, chemically synthesised or green ZnO nanoparticles; co-treatment	Improved hippocampal antioxidant, neurotrophin and histological outcomes, with formulation-dependent responses within the study.
Adelakun et al., 2022 [35]	Reproductive	Rats; cisplatin	Zinc sulphate; post-chemotherapy supplementation	Improved sperm parameters, reproductive hormones, antioxidant status and testicular structure.
Ali & Aziz, 2022 [36]	Nervous system	Rats; oxaliplatin	Zinc alone or with melatonin; pre-treatment/co-treatment or co-treatment only	Zinc alone provided partial protection. Zinc–melatonin combinations improved nociceptive and tissue outcomes; their effects cannot be attributed to zinc alone.
Hamam et al., 2022 [37]	Reproductive	Prepubertal rats; cisplatin	ZnO nanoparticles; pre-treatment and continued supplementation	Improved testicular structure, blood–testis barrier markers, sperm quality and sperm DNA integrity.
Majd et al., 2023 [38]	Kidney	Rats; repeated cisplatin	Bulk, chemically synthesised or green ZnO nanoparticles; co-treatment	Improved renal-function, oxidative-stress, apoptotic and histological outcomes, with formulation-dependent responses within the study.
Al-Lbban et al., 2024 [39]	Cardiovascular	Rats; cisplatin	ZnO nanoparticles; post-chemotherapy supplementation	Reported dose-dependent improvements in cardiac oxidative/inflammatory markers, lipid profiles and related biochemical outcomes.
Kotb et al., 2024 [40]	Liver	Rats; cisplatin	Chitosan-based ZnO nanocomposite; pre-treatment and co-treatment	Improved hepatic biochemical and histological outcomes, with reduced oxidative stress and inflammatory markers.

ZnO, zinc oxide.

**Table 2:** Summary of animal studies investigating zinc-containing interventions against BEP-associated reproductive toxicity.

Study	Organ/System	Animal Model and Chemotherapy	Zinc Formulation and Timing	Key Findings
Kilarkaje et al., 2013 [42]	Reproductive	Rats; three BEP cycles	Zinc-containing antioxidant cocktail; before chemotherapy and throughout treatment and recovery	Provided partial protection during treatment and improved reproductive recovery. Effects cannot be attributed to zinc independently of the other cocktail components.
Kilarkaje et al., 2014 [43]	Reproductive/endocrine	Rats; short-course or repeated-cycle BEP, with a recovery arm	Zinc-containing antioxidant cocktail; before and during chemotherapy, continued during recovery where applicable	Partially improved steroidogenesis and testicular recovery without complete restoration. Zinc-specific effects were not isolated.
Razavi et al., 2019 [44]	Reproductive	Rats; repeated-cycle BEP	Zinc sulphate; post-chemotherapy supplementation	Partially improved sperm parameters, chromatin condensation and testicular architecture; outcomes did not fully return to control levels.
Khadivi et al., 2019 [45]	Reproductive	Rats; repeated-cycle BEP	Zinc sulphate; post-chemotherapy supplementation	Improved sperm chromatin integrity and testicular histomorphometry, with incomplete recovery of some structural outcomes.

BEP, bleomycin–etoposide–cisplatin.

**Table 3:** Zinc interventions and antitumour outcomes in the included platinum chemotherapy studies.

Study	Tumour Model	Zinc Formulation, Dose and Timing	Platinum Regimen in the Tumour Experiment	Antitumour Outcomes and Findings
Doz et al., 1992 [41]	Intracerebral 9L gliosarcoma in Fischer 344 rats	High-zinc diet containing 180 ppm zinc, compared with a control diet containing 10 ppm, for 12–14 days before carboplatin administration	Carboplatin, 150 mg/kg i.p., single dose	Tumours were collected 24 h after treatment for an ex vivo clonogenic assay. Tumour cell fractional survival did not differ significantly between dietary groups in two replicate experiments. MT mRNA induction was not detected in the tumours.
Satoh et al., 1993 [25]	Subcutaneous MBT-2 bladder tumours in C3H mice	ZnSO_4_, 200 µmol/kg s.c. daily on days 8–9 after tumour inoculation; platinum administered on day 10. Additional groups received PPG, 500 µmol/kg s.c. daily on days 7–9	Cisplatin, 50 µmol/kg i.p., single dose on day 10	Tumour weight was assessed on day 14. Zinc-induced tumour MT was associated with reduced cisplatin antitumour activity. PPG suppressed tumour MT induction and restored antitumour activity while retaining renal protection.
Chan et al., 2019 [30]	Orthotopic SKNLP neuroblastoma xenografts in SCID Beige mice	BiZn, 0.14 mmol/kg orally, 24 h before and immediately before cisplatin, followed by administration every 2 days	Cisplatin, 7.5 mg/kg i.p. weekly for 3 weeks	Tumour volume and bioluminescence, including signals at lung and liver metastatic sites, were evaluated. No significant impairment of cisplatin-mediated tumour control was detected with BiZn.

Platinum regimens refer to the tumour experiments. Tumour cell fractional survival denotes clonogenic survival, not animal survival. BiZn, bismuth zinc citrate; i.p., intraperitoneal; MT, metallothionein; PPG, propargylglycine; s.c., subcutaneous.

## Data Availability

This article is a systematic review of previously published studies. It therefore contains no primary datasets generated or analysed during the current study.

## Supplementary Materials

The following supporting information can be downloaded at: https://www.mdpi.com/article/10.3390/ijms27219438/s1.
