# Supplementary material for: Platinum-Induced Toxicity and Zinc-Based Interventions: A Systematic Review of Preclinical Evidence
Source: Int J Mol Sci. 2026 Oct 23;27(21):9438. doi: 10.3390/ijms27219438 (PMC13763258; doi:10.3390/ijms27219438)
Supplement: Supplementary file 1 [file ijms-27-09438-s001.zip › ijms-4616765-supplementary.pdf]

Supplementary Table S1: Search strategies and retrieval counts for PubMed, Scopus and Web of Science (11th of June 2026).

Search	Query	Items found
**PubMed**		
#1	Search: zinc OR zinc[tw] OR "Zinc"[Mesh]	210,388
#2	Search: cisplatin OR cisplatin[tw] OR oxaliplatin OR oxaliplatin[tw] OR carboplatin OR carboplatin[tw] OR "platinum-based" OR platinum OR platinum [tw] OR platin* OR "Cisplatin"[Mesh] OR "Oxaliplatin"[Mesh] OR "Carboplatin"[Mesh]	203,081
#3	Search: rats OR rat OR mouse OR mice OR murine OR rodent OR rodents OR rodentia OR "Rats"[Mesh] OR "Mice"[Mesh] OR "Rodentia"[Mesh]	4,319,788
#4	#1 AND #2 AND #3	279
**Scopus**		
#1	(rats OR rat OR mouse OR mice OR murine OR rodent OR rodents OR rodentia) AND (cisplatin OR oxaliplatin OR carboplatin OR "platinum-based" OR platinum OR platin*) AND zinc	661
**Web of Science**		
#1	(rats OR rat OR mouse OR mice OR murine OR rodent OR rodents OR rodentia) AND (cisplatin OR oxaliplatin OR carboplatin OR "platinum-based" OR platinum OR platin*) AND zinc	612

Supplementary Table S2: Supplementary search strategies, reported result counts and screening limits for Google Scholar and ProQuest (11th of June 2026).

Search	Query	Items found	Records screened
Google Scholar	zinc AND (cisplatin OR oxaliplatin OR carboplatin) AND (rats OR rat OR mouse OR mice OR murine OR rodent OR rodents OR rodentia)	"about 84,400 results"	1,000
ProQuest	zinc AND (cisplatin OR oxaliplatin OR carboplatin) AND (rats OR rat OR mouse OR mice OR murine OR rodent OR rodents OR rodentia)	33,813	1,000

Supplementary Table S3: Detailed characteristics, intervention regimens and outcomes of studies investigating zinc-based interventions against toxicity induced by platinum agents alone.

Zn, zinc; CIS, cisplatin [cis-diamminedichloroplatinum(II)]; ZnAc, zinc acetate; $CuSO_4$, copper sulfate; FSH, follicle-stimulating hormone; LH, luteinizing hormone; ABP, androgen-binding protein; i.p., intraperitoneal; s.c., subcutaneous; p.o., per os (referring to oral gavage); NS, not significant; MT, metallothionein; SOD, superoxide dismutase; TBA, thiobarbituric acid; MDAeq, malondialdehyde equivalents; MDA, malondialdehyde; $ZnCO_3$, zinc

**Study**	**Organ/system studied**	**Animal model of platinum-induced toxicity**	**Zinc intervention (form, dose, route, schedule)**	**Experimental groups (n per group)**	**Main findings**
Pogach et al., 1989	Reproductive (hormonal axis: LH/FSH/testosterone, Sertoli ABP, Leydig & pituitary function)	Adult male Sprague-Dawley rats;<br>CIS 2 mg/kg i.p. daily for 5 days; sacrifice 1 week after first dose	ZnAc, 6 mg/kg/day i.p., 5 days before CIS, continued during CIS (pretreatment + co-treatment; total 10 days)	• Negative controls<br>• ZnAc only<br>• $CuSO_4$ only<br>• Positive controls (CIS-only)<br>• CIS+ZnAc<br>• CIS+$CuSO_4$ (n = 5/group)	• CIS caused ↓LH, ↓FSH, ↓serum testosterone (–77%), ↓testicular testosterone (–82%), ↓testicular ABP (–57%).<br>• ZnAc did not protect LH/FSH or testosterone levels; ZnAc alone also reduced testicular T.<br>• ZnAc partially prevented Sertoli cell dysfunction: testicular ABP only –15% vs control (NS), vs –57% with CIS alone.<br>• No protection for Leydig or pituitary function.
Reeves et al., 1990	Kidney (nephrotoxicity: serum urea, markers of oxidative stress, MT induction); liver trace element metabolism.	Young male Sprague-Dawley rats (~88 g);<br>Single bolus CIS administration (7.5 mg/kg i.p.);<br>Tissues collected 3 days after injection	Dietary zinc (as $ZnCO_3$) at 5, 20, 80, or 320 mg Zn/kg diet, fed for 3 weeks prior to CIS and maintained thereafter	Four dietary Zn levels × 3 treatment arms:<br>• Negative controls<br>• Positive controls (CIS-only) (7.5 mg/kg i.p. once)<br>• Pair-fed saline (matched to CIS food intake) (n = 7/group)	• Higher dietary Zn increased baseline kidney/liver MT; CIS further increased kidney MT at all Zn levels.<br>• High dietary Zn did not reduce CIS nephrotoxicity: CIS raised serum urea and decreased body weight similarly at all Zn levels.<br>• High Zn caused mild copper deficiency (↓ serum Cu, ↓ ceruloplasmin), likely masking any protective MT effect.<br>• CIS reduced kidney SOD slightly (~7%) and increased lipid peroxidation (MDAeq).<br>• Higher dietary Zn reduced baseline lipid peroxidation but did not mitigate CIS-induced renal injury.

Doz et al., 1992	Bone marrow (hematotoxicity: CFU-GM); Kidney (MT mRNA induction as mechanistic marker of systemic platinum toxicity)	DBA/2 mice, 6 weeks old (~20 g); CARB given i.p. at 150–300 mg/kg (single dose); Bone marrow collected 24 h post-carboplatin for CFU-GM assay (myelotoxicity)	High-zinc diet (180 ppm zinc p.o.) vs control diet (10 ppm zinc p.o.), 12–14 days prior to CARB administration	• Control diet + HBSS<br>• Control diet + CARB<br>• High-zinc diet + HBSS<br>• High-zinc diet + CARB<br>(n = 4-6/group)	• High-zinc diet did not alter baseline CFU-GM in untreated mice.<br>• High-zinc diet reduced CARB hematotoxicity: At 180 mg/kg CARB, CFU-GM survival was higher compared to control-diet mice, allowing for 50% CARB dose escalation (up to 225 mg/kg) without additional myelotoxicity.<br>• Significant induction of MT mRNA in kidney and bone marrow on the high-zinc diet, supporting MT-mediated protection of hematopoietic tissue.<br>• Dietary zinc pre-exposure increased the bone-marrow tolerance to CARB, suggesting a higher therapeutic window from a toxicity standpoint.
Satoh et al., 1993	Kidney (nephrotoxicity; MT induction, nephroprotection, antitumor response modulation)	Female C3H mice, 5 weeks old, inoculated s.c. with 1×10^6 MBT-2 bladder tumor cells (day 0); CIS 50 µmol/kg i.p. given on day 10; Renal toxicity assessed on day 14 (4 days post-CIS)	$ZnSO_4$, 200 µmol/kg s.c., once daily on days 8-9 (2 days before CIS)	• Negative controls (tumor-bearing)<br>• $ZnSO_4$<br>• $ZnSO_4$ + PPG<br>• Positive controls (CIS-only)<br>• $ZnSO_4$ + CIS<br>• PPG + CIS<br>• $ZnSO_4$ + PPG + CIS<br>(n = 4/per group)	• $ZnSO_4$ increased MT in tumor, kidney, and liver.<br>• Zinc pretreatment protected against CIS nephrotoxicity: CIS ↑BUN markedly; $ZnSO_4$ pretreatment kept BUN at control levels.<br>• $ZnSO_4$ reduced CIS antitumor activity (lower tumor response due to high tumor MT).<br>• PPG blocked MT induction in tumor but not kidney, so CIS antitumor activity was restored while renal protection was maintained.<br>• Zinc alone did not alter tumor growth or renal function.
Nakagawa et al., 1996	Kidney (nephrotoxicity, oxidative stress markers: BUN, TBA-RS, renal GSH)	Male CD-1 (ICR) mice, 5 weeks old; CIS administered s.c. as a single dose of 45 µmol/kg, 24 h after last $ZnCl_2$ administration;	$ZnCl_2$, 200 µmol/kg s.c., once daily for 2 days, with CIS given 24 h after last zinc dose	• Negative controls<br>• BSO only<br>• Positive controls (CIS-only)<br>• BSO + CIS	• CIS alone did not significantly increase BUN or TBA-RS (confirmation of subtoxic dose).<br>• BSO-mediated GSH depletion dramatically enhanced CIS nephrotoxicity, causing marked increases in BUN and renal lipid peroxidation (TBA-RS).<br>• $ZnCl_2$ pretreatment strongly induced renal MT (≈13-fold over control at time of CIS injection).

		Glutathione depletion model: BSO (4.5 mmol/kg s.c.) given 4 h before CIS; Renal toxicity assessed 12-48 h post-CIS (BUN, TBA-RS, renal GSH)		• $ZnCl_2$ only (2-day MT induction)<br>• $ZnCl_2$ + BSO<br>• $ZnCl_2$ + CIS<br>• $ZnCl_2$ + BSO + CIS (n = 4-5/group, depending on endpoint)	• $ZnCl_2$ pretreatment attenuated the BSO-enhanced nephrotoxicity:<br>– prevented the CIS-induced rise in BUN<br>– blocked the CIS-induced rise in TBA-RS at 12 h<br>– improved recovery of renal GSH.<br>• No increase in Pt bound to MT on chromatography, despite high MT levels; instead MT levels dropped after CIS, consistent with oxidation of MT thiols.<br>• Conclusion that MT protects against CIS toxicity via free-radical scavenging, not via platinum binding, particularly in GSH-depleted states.
Huang et al., 1997	Kidney (nephrotoxicity: serum Cr, BUN, renal GSH, renal MDA, GSH-Px, CAT)	Kunming mice, ~21 ± 2 g; Single CIS administration (9 mg/kg i.p.), on day 6 (after 5 days of pretreatment); Sacrifice: 48 h after CIS administration	ZG aministered p.o. in a dose of 100, 140, or 180 mg/kg/day, once daily for 7 days	• Negative controls (saline p.o. for 7 days + saline i.p.)<br>• Positive controls (saline p.o. for 7 days + CIS)<br>• ZG 100 mg/kg + CDDP<br>• ZG 140 mg/kg + CIS<br>• ZG 180 mg/kg + CIS<br>(n = 10/group)	• ZG 140 and 180 mg/kg significantly decreased serum Cr and BUN and renal MDA compared with the CDDP group.<br>• ZG 140 and 180 mg/kg increased whole-blood GSH levels.<br>• ZG had minimal effect on GSH-Px and CAT in renal tissue.<br>• ZG attenuated cisplatin nephrotoxicity via improved glutathione metabolism and reduced lipid peroxidation.
Satoh et al., 1997	Kidney (nephrotoxicity: BUN, plasma creatinine, histopathology)	Female mice; MT-null mice (MT-I/II knockout; mixed 129 Ola × C57BL/6	$ZnSO_4$ 100 µmol/kg s.c., once daily for 2 days, then CIS administered 24 h after last zinc dose	<u>WT (C57BL/6J):</u><br>• Control (saline)<br>• CIS 30 µmol/kg<br>• CIS 40 µmol/kg	• MT-null mice were dramatically more sensitive to cisplatin nephrotoxicity (higher BUN and Cr at every cisplatin dose tested and severe tubular degeneration and necrosis at 40 µmol/kg, much worse than WT.

		background) and WT controls (female C57BL/6J); Single dose CIS 30-60 µmol/kg i.p.		• $ZnSO_4$, then CIS 30 µmol/kg<br>• $ZnSO_4$, then CIS 40 µmol/kg<br>MT-null mice:<br>• Control<br>• CIS 30 µmol/kg<br>• CIS 40 µmol/kg<br>• $ZnSO_4$, then CIS 30 µmol/kg<br>• $ZnSO_4$, then CIS 40 µmol/kg (renal outcomes: n = 4/group; survival: n = 7/group)	• In WT mice CIS (40 µmol/kg) significantly elevated BUN and creatinine. $ZnSO_4$ pretreatment induced high renal MT levels and completely prevented the BUN and Cr rise.<br>• Histologically marked reduction of tubular injury in zinc-pretreated animals.<br>• In MT-null mice, $ZnSO_4$ pretreatment failed to raise MT and offered no protection; BUN, Cr, and histopathology remained severely abnormal.
Satoh et al., 2000	Kidney (nephrotoxicity: BUN, serum creatinine; histopathology; renal MT and GSH levels)	Female MT-null mice (MT-I/II knockout, 129 Ola × C57BL/6 background, backcrossed to C57BL/6);<br>Female WT ($MT^{+}/^{+}$) littermates as controls; age: 8 weeks old;<br>CIS 30 µmol/kg i.p., single dose.	$ZnSO_4$ 200 µmol/kg s.c., once daily for 2 days	WT:<br>• Negative controls<br>• WT + CIS<br>• WT + BSO, then CIS<br>• WT + Zn, then CIS<br>• WT + Zn, then BSO, then CIS<br>MT-null counterparts:<br>• MT-null control<br>• MT-null + CIS<br>• MT-null + BSO, then CIS	• MT-null mice were markedly more sensitive to cisplatin nephrotoxicity (higher BUN/Cr; severe tubular necrosis)<br>• GSH depletion (BSO) dramatically worsened cisplatin renal toxicity in both genotypes<br>• Combined MT deficiency + GSH depletion produced the most severe nephrotoxicity, with extensive tubular necrosis<br>• Zinc pretreatment protected WT mice from BSO-enhanced cisplatin toxicity by preinducing MT, reducing BUN/Cr and histological damage<br>• Zinc pretreatment provided no protection in MT-null mice, confirming that MT induction is required for zinc's nephroprotective effect

		Glutathione depletion model: BSO 2.5 mmol/kg s.c., given 4 h before CIS to reduce renal GSH. Renal toxicity assessed 2 days after CIS		• MT-null + Zn, then CIS<br>• MT-null + Zn, then BSO, then CIS (n = 4/group)	
Liao et al., 2008	Liver (hepatotoxicity: ALT, hepatic GSH, hepatic MDA, liver ratio, weight loss)	Male albino mice, ~30 g;<br>CIS 3.5 mg/kg i.p. once daily for 5 days;<br>Toxicity evaluated after completion of a 9-day supplementation + CIS course	$ZnSO_4$, 0 or 25 mg/kg/day p.o., given once daily for 9 days (2 days before CIS; continued during and after treatment according to orthogonal design)	Orthogonal design L8(2^7) → 8 combinations of the 7 factors (with 5 mice/group):<br>• $ZnSO_4$: 0 or 25 mg/kg/day p.o.<br>• Na_2SeO_3: 0 or 1.5 mg/kg/day p.o.<br>• Fos: 0 or 300 mg/kg/day p.o.<br>• NAC: 0 or 500 mg/kg/day p.o.<br>• STS: 0 or 1000 mg/kg/day p.o.<br>• Met: 0 or 200 mg/kg/day p.o.<br>• Tau: 0 or 400 mg/kg/day p.o.<br>• Control groups included: CIS-only	• CIS alone caused clear hepatotoxicity: ↑ALT, ↑MDA, ↓GSH, ↓liver ratio, and marked weight loss<br>• No single agent was sufficient to completely prevent hepatotoxicity<br>• Fos improved nearly all markers of liver injury (weight loss, liver ratio, ALT, MDA)<br>• Met was the only agent that significantly preserved hepatic GSH<br>• Selenium and zinc reduced oxidative stress (lower MDA) and improved ALT<br>• STS helped reduce ALT elevations<br>• Tau and NAC showed little or no protective effect on hepatic endpoints<br>• Best-performing combinations (most hepatoprotective) were:<br>→ Zn(0) + Se(1) + Fos(1) + NAC(0) + STS(0) + Met(1) + Tau(1)<br>→ Zn(1) + Se(1) + Fos(0) + NAC(0) + STS(2) + Met(2) + Tau(1)

				group (baseline toxicity); healthy control (vehicle only)	
Tuzcu et al., 2010	Kidney (nephrotoxicity: renal function, oxidative stress, inflammation, apoptosis, stress proteins)	Male Wistar rats, 8 weeks old, 200-220 g;<br>Single dose CIS 7 mg/kg i.p.; Toxicity assessed 10 days later	Zinc picolinate; 6 mg elemental Zn/kg/day i.p. once daily for 10 days (before and after CIS)	• Negative controls<br>• Zinc picolinate only<br>• Positive controls (CIS-only)<br>• CIS + zinc picolinate<br>(n = 7/group)	• CIS alone: marked ↑urea-N, ↑creatinine, ↑MDA, ↑8-isoprostane, ↑TNF-α, ↑Bax, ↑HSP60/70, ↓kidney Zn<br>• Zinc picolinate significantly improved renal function (↓creatinine, ↓urea-N), with strong reduction in oxidative stress (↓MDA, ↓8-isoprostane) and inflammation (↓TNF-α), beneficial anti-apoptotic shift (↓Bax, ↑Bcl-2), lower expression of HSP60 and HSP70, and restored kidney zinc levels; no effect on weight loss
Chan et al., 2019	Kidney (nephrotoxicity: BUN, creatinine, renal tubular injury, survival)	Male mice (SCID Beige and BALB/c);<br>• Standard-dose CIS: 7.5 mg/kg × 2 + 15 mg/kg (21-day nephrotoxicity protocol)<br>• Lethal-dose CIS: 15 mg/kg × 2 (survival model)<br>• Orthotopic SKNLP neuroblastoma xenograft to assess BiZn effects on CIS	BiZn 0.14 mmol/kg p.o. given 24 h before CIS, again just prior to CIS, and then every 2 days; Safety also tested at 0.58 mmol/kg for 5 days (short-term toxicity assay)	<u>Toxicity study (SCID Beige mice):</u><br>• Negative controls<br>• Positive controls (CIS-only)<br>• BiZn alone<br>• BiZn + CIS<br>(n = 6/group)<br><u>Lethal-dose survival study:</u><br>• CIS high-dose alone<br>• BiZn + CIS high-dose<br>(n = 10/group)<br><u>BiZn safety study (BALB/c mice):</u><br>BiZn only, 0.58	• BiZn significantly reduced nephrotoxicity:<br>↓BUN and creatinine vs CIS alone<br>↓tubular damage on histology (↓vacuolation, casts, epithelial desquamation)<br>• BiZn improved survival under lethal cisplatin dosing<br>• BiZn increased renal MT and GSH, whereas MT-IIA knockdown or GSH inhibition abolished protection<br>• BiZn did not affect CIS tumor control in the neuroblastoma xenograft (no promotion of tumor growth; no interaction with the antitumor effect of CIS)<br>• BiZn alone: no organ toxicity at tested doses.

		antitumor efficacy		mmol/kg, 5 days (n = 5)	
Barakat et al., 2020	Kidney (nephrotoxicity; renal function, oxidative stress, apoptosis/necrosis, inflammation, histological tubular damage and regeneration)	Adult male Sprague-Dawley rats (280-330 g); Single dose CIS 6 mg/kg i.p. for acute kidney injury induction; Outcomes assessed at 7- and 12-days post-injection	Pre-treatment with ZnO-NPs, single dose 5 mg/kg, i.p., given 2h before CIS (particle size ≤40 nm, purity >97%); Separate ZnO-NPs-only group received 5 mg/kg IP without CIS	• Negative controls<br>• ZnO-NPs only<br>• Positive controls (CIS-only)<br>• ZnO-NPs + then CIS (2h later)<br>(n = 12/group)	• CIS caused marked nephrotoxicity, shown by ↑serum Cr, BUN, and microalbuminuria, ↑renal MDA, ↓SOD, CAT, and GSH, and extensive tubular injury (necrosis, dilation, casts, interstitial fibrosis)<br>• Pre-treatment with ZnO-NPs (5 mg/kg, i.p.) significantly attenuated kidney injury, normalizing kidney function markers and improving oxidative stress status (↓MDA, ↑SOD/CAT/GSH).<br>• ZnO-NPs: ↓apoptosis and necrosis (Annexin V), ↓TGF-β1, anti-inflammatory effects, restored protective gene expression (↑Nrf2, ↑HO-1, ↑eNOS; ↓Bax) and improved histopathology, with ↓tubular damage and ↑regenerative Ki-67 staining.
Shakibaie et al., 2020	Reproductive (testicular toxicity; oxidative stress; histopathology; spermatogenesis index)	NMRI male mice; Single dose CIS 7 mg/kg i.p.on day 10; Toxicity assessment on day 15	Zn NPs (synthesis via microwave-assisted Lavandula vera extract method); doses: 10, 25, 50 mg/kg, p.o., daily for 14 days; Comparator zinc form: $ZnSO_4$, 10 mg/kg, oral, daily for 14 days)	• Negative controls<br>• Positive controls (CIS-only)<br>• $ZnSO_4$ + CIS<br>• Zn NP 10 mg/kg + CIS<br>• Zn NP 25 mg/kg + CIS<br>• Zn NP 50 mg/kg + CIS<br>• Vitamin E + CIS (active antioxidant comparator)<br>(n = 6/group)	• CIS caused marked testicular oxidative stress (↑MDA, ↓GSH, ↓SOD/CAT) and severe seminiferous epithelial damage with impaired spermatogenesis.<br>• Zn NPs improved oxidative stress parameters in a dose-dependent manner (↓MDA, ↑GSH/SOD/CAT).<br>• High-dose Zn NPs (50 mg/kg) provided the strongest protection, achieving near-normal oxidative marker levels and mild histopathological damage, comparable to vitamin E.<br>• $ZnSO_4$ (10 mg/kg) improved oxidative stress but was less protective histologically than Zn NPs.

Majd et al., 2021a	Reproductive (testicular histology; sperm parameters; oxidative stress; testosterone; steroidogenesis gene expression)	Male Wistar rats (8-9 weeks, 250-270 g);<br>CIS 5 mg/kg/week i.p. for 4 weeks;<br>ZnO co-treatment given concurrently;<br>Outcome assessment at end of week 4	• ZnO bulk (Zn-B); 5 mg/kg/day i.p.<br>• Green ZnO-NPs (ZnG-NPs): Aloe vera-mediated "green" ZnO-NPs, ~20-50 nm (TEM), mean size ~36 nm (PSA); 5 mg/kg/day i.p.<br>• Chemical ZnO NPs (ZnC-NPs): commercial ZnO-NPs; 5 mg/kg/day i.p.	• Negative controls<br>• Sham (handling/environ. stress)<br>• Zn-B only<br>• ZnG-NP only<br>• ZnC-NP only<br>• Positive controls (CIS-only)<br>• CIS + Zn-B<br>• CIS + ZnG-NP<br>• CIS + ZnC-NP (n = 5/group)	• CIS caused marked testicular toxicity: reduced body/testis weight, severe seminiferous tubule damage, fibrotic changes, impaired spermatogenesis, poor sperm parameters, high oxidative stress and reduced testosterone with suppressed steroidogenesis<br>• ZnG-NPs provided the strongest protection, significantly improving testis morphology and seminiferous architecture, sperm count, motility, morphology and viability, antioxidant enzyme activity (↑SOD/GPX/CAT), serum testosterone, STAR gene expression, overall preservation of spermatogenesis<br>• ZnC-NPs showed moderate protection, improving oxidative stress, testosterone, STAR expression, and several sperm parameters, but less effectively than green ZnO NPs.<br>• ZnO bulk showed weak protection, with minimal improvement in sperm parameters or steroidogenesis.
Majd et al., 2021b	Central nervous system (hippocampal neurotoxicity – oxidative stress, neurotrophins, histology, histomorphometry)	Adult male Wistar rats;<br>CIS 5 mg/kg i.p., once weekly for 4 weeks;<br>Assessment at the end of week 4 (day 28)	• Zn-B: 5 mg/kg/day i.p.<br>• ZnC-NPs: 5 mg/kg/day i.p. (20–30 nm, commercial)<br>• ZnG-NPs: Aloe vera-mediated "green" ZnO-NPs: 5 mg/kg/day i.p.; size: mostly spherical, ~20–	• Negative controls<br>• Sham (handling/environ. stress)<br>• ZnC-NPs<br>• ZnG-NPs<br>• Positive controls (CIS-only)<br>• CIS + ZnOB<br>• CIS + ChZnO NPs<br>• CIS + GrZnO NPs<br>• CIS + Aloe vera extract (5	• CIS caused marked hippocampal oxidative stress (↓SOD, ↓CAT, ↓GPx, ↓GSH, ↑GSSG, ↓GSH/GSSG ratio, ↑MDA)<br>• Green ZnO NPs (ZnG-NPs) provided the strongest protection, by normalizing antioxidant enzymes, restoring glutathione balance, reducing lipid peroxidation, restoring NGF and BDNF mRNA expression, improving neuronal morphology and counts in CA1/CA3/DG, and increasing pyramidal/granular layer thickness.<br>• ZnC-NPs showed moderate antioxidant improvement (increased NGF and BDNF expression and partial histological recovery)

			50 nm by TEM; PSA: 10–65 nm, mean ~36 nm)<br>Zn was given daily for 28 days	mg/kg/day) (n = 8/group)	• Zn-B had weak antioxidant effects with minimal histological protection.
Adelakun et al., 2022	Reproductive system (spermatogonial and testicular toxicity; sperm parameters; oxidative stress; histology; morphometry; daily sperm production)	Adult male Sprague-Dawley rats (8 weeks old, ~150 g); Single dose CIS 10 mg/kg i.p., testicular toxicity assessed at 8 weeks (full spermatogenic cycle)	• $ZnSO_4 \cdot 7H_2O$ 1 mg/kg/day, p.o., for 8 weeks in 2 regimens:<br>– Preventive Zn-only group<br>– Therapeutic post-CIS supplementation in CP+Zn group	• Negative controls<br>• Zn-only<br>• Positive controls (CIS-only)<br>• CIS + Zn<br>(n = 8/group)	• CIS caused severe testicular toxicity: ↓testis weight/volume, ↓sperm count/motility/morphology, ↓DSP/EDSP, ↓testosterone/FSH/LH, ↓CAT/SOD/POD/GR, ↑TBARS, seminiferous epithelial damage, ↓germ cells, ↓Johnsen score.<br>• $ZnSO_4$ significantly restored sperm parameters, DSP, testicular morphometry, germ cell numbers, and endocrine hormones toward control.<br>• $ZnSO_4$ improved antioxidant status (↑CAT/SOD/POD/GR, ↓TBARS), with preserved seminiferous epithelium, reduced fibrosis, and improved Johnsen score on histology
Ali & Aziz, 2022	Central nervous system (neurotoxicity and peripheral neuropathy – thermal nociception; oxidative stress; inflammatory markers; histology; immunohistochemistry)	Male Wistar albino rats (150-200 g); OXL 4 mg/kg i.p. on days 1, 2, 5, and 6 (total cumulative dose 16 mg/kg); Neurotoxicity/neurobehavioral and tissue endpoints assessed 24 h after last OXL dose	Zinc 15 mg/kg/day p.o. in 2 regimens:<br>• Prevention regimen (Zn, Mel, Zn+Mel prevention groups): daily for 2 weeks before OXL, then continued daily for 1 week during OXL protocol	• Negative controls<br>• Positive controls (OXL-only)<br>• Zn + OXL (prevention)<br>• Melatonin + OXL (prevention): melatonin 10 mg/kg/day p.o. (same schedule)<br>• Zn + Mel + OXL (prevention**)**	• OXL induced neurotoxicity: thermal hyperalgesia & cold allodynia, ↓GPX/SOD, ↑GFAP, ↑NF-κB & TNF-α, severe cerebral/cerebellar lesions (edema, hemorrhage, microthrombi, pyknotic cells), ↑p53 and ↓Bcl-2.<br>• Zinc alone: partial improvement in nociceptive responses, ↓GFAP, mild antioxidant restoration, modest histological protection.<br>• Mel alone: similar partial protection (↑GPX/SOD, ↓GFAP), limited anti-inflammatory effect.<br>• Zinc + Mel (prevention regimen): strongest protection—normalised hot/cold nociception, significant ↑GPX/SOD, marked ↓GFAP, lowest lesion scores, and strong ↓p53 expression.

			• Treatment regimen (Zn+Mel treatment group): daily for 1 week during OXL protocol only (no pre-treatment)	• Zn + Mel + OXL (treatment – 1 week during OXL only)<br>(n = 8/group)	• Zinc + Mel (treatment regimen): improved nociception and ↓TNF-α/p53, with moderate histological improvement (less effective than prevention).
Hamam et al., 2022	Reproductive (testis – biochemistry, histology, immunohistochemistry, ultrastructure; spermatogenesis / BTB integrity; sperm quality; sperm DNA integrity; oxidative stress and inflammation; apoptosis	Prepubertal male Sprague-Dawley rats, 30 days old, 60-70 g;<br>Single CIS 5 mg/kg i.p.;<br>Sacrifice at 7, 30, 60 days after CIS, corresponding ages 37 d (prepubertal), 60 d (productive), 90 d (adult)	Commercial ZnO-NPs, <50 nm, surface area >10.8 m^2/g, 5 mg/kg i.p.<br>• ZnO-NPs group: once weekly for 8 weeks<br>• ZnO-NPs + CIS group: first dose of ZnO-NPs 2 h before CIS, then once weekly for 8 weeks	• Negative controls<br>• ZnO-NPs only<br>• Positive controls (CIS-only) at 30 days old<br>• ZnO-NPs + CIS<br>(n = 18/group; 6 per timepoint)	• CIS toxicity: ↓body/testis weight; ↑MDA; ↓CAT; BTB disruption (↓vimentin/occludin); ↓Bcl-2; ↑IL-6; severe testicular histopathology; ↓sperm count/motility/viability; ↑abnormal forms; ↑sperm DNA damage; ↓protamine.<br>• ZnO-NPs alone: No testicular toxicity; normal oxidative status, histology, and sperm quality.<br>• ZnO-NPs + CIS:<br>↓Oxidative stress (↓MDA; ↑CAT vs CIS)<br>↑BTB integrity: ↑vimentin & occludin (gene/protein)<br>regulation of apoptosis/inflammation: ↑Bcl-2; ↓IL-6, improved testis structure, and spermatogenesis (↑sperm count/motility/viability; ↓abnormal morphology)<br>DNA integrity: ↓comet tail length/moment; ↑protamine expression.

Majd et al., 2023	Kidney (nephrotoxicity: oxidative stress, apoptosis markers, renal function, histopathology)	Adult male Wistar rats (250-270 g);<br>CIS 5 mg/kg i.p., once weekly for 4 weeks;<br>Toxicity assessed on day 30	• Zn-B: 5 mg/kg/day, i.p., 4 weeks<br>• ZnC-NPs: 5 mg/kg/day, i.p., 4 weeks<br>• ZnG-NPs; Aloe vera–derived, ~20–50 nm): 5 mg/kg/day, i.p., 4 weeks	• Negative controls<br>• Sham (handling/environ. stress)<br>• Zn-B<br>• ZnC-NPs<br>• ZnG-NPs<br>• Positive controls (CIS-only)<br>• CIS + Zn-B<br>• CIS + ZnC-NPs<br>• CIS + ZnG-NPs<br>• CIS + Aloe vera extract (plant extract control)<br>(n = 8/group)	• CIS caused nephrotoxicity: ↑BUN, ↑MDA, ↓SOD/CAT, ↑bax, ↓bcl2, and severe tubular/glomerular injury.<br>• All ZnO forms reduced CIS nephrotoxicity (↓BUN, ↓MDA; partial restoration of SOD/CAT; ↓bax and ↑bcl2; improved histology).<br>• ZnG-NPs showed strongest protection across oxidative stress, apoptosis markers, renal function, and tissue injury.<br>• ZnC-NPs moderate benefit; Zn-B least effective but still protective.
Al-Lbban et al., 2024	Cardiovascular system (cardiotoxicity – serum lipid profile; atherogenic indices; kidney function; cardiac oxidant/antioxidant status; inflammatory markers)	Male albino rats, 140 ± 10 g;<br>Single CIS 20 mg/kg i.p.	ZnO-NPs administered p.o. in doses of 10, 20, 30, 40, 50 mg/kg/day for 15 consecutive days after CIS	• Negative controls<br>• Positive controls (CIS-only)<br>• CIS + ZnONPs 10 mg/kg<br>• CIS + ZnONPs 20 mg/kg<br>• CIS + ZnONPs 30 mg/kg<br>• CIS + ZnONPs 40 mg/kg<br>• CIS + ZnONPs 50 mg/kg<br>(n = 6/group)	• CIS cardiotoxicity (vs negative control) shown in: lipid profile, atherogenic indices, kidney function, cardiac oxidative stress, inflammation, and cardiac injury markers<br>• ZnONPs (10–50 mg/kg) + CIS: dose-dependent protection; progressive improvement in lipid profile with increasing ZnONPs dose ,improved atherogenic indices, most normalized at 50 mg/kg, improved renal markers, approaching control at higher doses, antioxidant defense restored in heart tissue, inflammation reduced (near control levels at 50 mg/kg), cardiac injury markers reduced

Kotb et al., 2024	Liver (hepatotoxicity – serum liver function tests; hepatic oxidative stress markers; inflammatory cytokine TNF-α; liver histopathology; immunohistochemistry for TGF-β1 and Bcl-2)	Male Sprague–Dawley albino rats, 6-8 weeks, 120-140 g;<br>Single CIS 12 mg/kg i.p. on day 10	QACSB–ZnO NCs (ZnO NPs encapsulated in a quaternary ammonium chitosan Schiff base; mean particle diameter ≈80 nm, zeta potential ≈+39.6 mV); 150 mg/kg/day i.p. for 14 days	• Negative controls<br>• Positive controls (CIS-only)<br>• QACSB-ZnO NCs + CIS: QACSB-ZnO NCs 150 mg/kg/day i.p. for 14 days + single CIS 12 mg/kg i.p. on day 10, 1 h after QACSB-ZnO (n = 10/group)	• CIS hepatotoxicity (vs control): ↑ALT/AST/ALP, ↓albumin/total protein, ↑MDA, ↓GSH, ↑TNF-α, severe hepatic degeneration and inflammation, ↑TGF-β1, ↓Bcl-2.<br>• QACSB-ZnO + CIS: improved liver enzymes, ↑albumin/total protein, ↓MDA, ↑GSH, ↓TNF-α, preserved liver histology, ↓TGF-β1, ↑Bcl-2.

Supplementary Table S4: Detailed characteristics, intervention regimens and outcomes of studies investigating zinc-containing interventions against BEP-associated reproductive toxicity.

Abbreviations: AC, antioxidant cocktail; BEP, bleomycin-etoposide-cisplatin; CIS, cisplatin; i.p., intraperitoneal; p.o., oral administration; mg/kg, milligrams per kilogram body weight; $ZnSO_4 \cdot 7H_2O$, zinc sulfate heptahydrate.

Study	Organ/system studied and outcomes	Animal model of chemotherapy-induced toxicity	Zinc intervention (form, dose, route, schedule)	Experimental groups (n per group)	Main findings
Kilarkaje et al., 2013	Reproductive (testes, sperm function, fertility, pituitary-testicular axis)	Adult male Sprague-Dawley rats (13-15 weeks); Three 21-day cycles of BEP chemotherapy:<br>• Bleomycin 0.75 mg/kg<br>• Etoposide 7.5 mg/kg<br>• CIS 1.5 mg/kg	Zinc given daily (as part of an AC), starting 1 week before BEP and continued through 9-week recovery;<br>AC included:<br>• Vitamin E 100 mg/kg (oral)<br>• Vitamin C 50 mg/kg (oral)<br>• Zinc 40 mg/L in drinking water<br>• Selenium 100 µg/L	• Negative controls (saline)<br>• AC only<br>• Positive controls (BEP-only)<br>• BEP + AC<br>(n = 10-15/group)	• BEP caused profound testicular toxicity: ↓sperm count, ↓motility, abnormal morphology, tubular atrophy, impaired fertility, disrupted hormones.<br>• Antioxidant cocktail provided partial acute protection: better sperm motility and morphology without prevention of severe testicular injury during treatment.<br>• During 63-day recovery, antioxidants improved recovery of sperm count and fertility and germ-cell repopulation, supported recovery of Leydig/Sertoli cell function, and normalized hormonal disturbances
Kilarkaje et al., 2014	Reproductive (testes and testosterone synthesis pathway – steroidogenic enzymes in Leydig cells; endocrine/testicular toxicity)	Adult male Sprague–Dawley rats (13-15 weeks);<br>• Short-term BEP, 4-day course (1× human-equivalent dose); Bleomycin 1.5	Zinc given daily (as part of an AC), starting 1 week before BEP and continued during the 9-week recovery in the recovery arm (zinc 40 mg/L in drinking water);<br>Other AC components included:	In each of the three experiments there were<br>• Controls<br>• AC only<br>• Positive controls (BEP-only)<br>• BEP + AC<br>(n = 3-6/group)	• BEP caused clear testicular toxicity, especially with long-term exposure: ↓testosterone production, disruption of the testosterone synthesis pathway, persistent impairment even after treatment cycles<br>• Short-term BEP produced minimal toxicity without significantly changing testosterone levels<br>• AC partially reduced BEP testicular toxicity, improved testosterone synthesis, provided

		mg/kg i.p. (day 2),<br>Etoposide 15 mg/kg i.p. (days 1-4),<br>CIS 3 mg/kg i.p. (days 1-4)<br>• Three 21-day cycles of BEP (as in clinical protocols) (0.5× dose; "clinical-type" protocol); Bleomycin 0.75 mg/kg i.p., Etoposide 7.5 mg/kg i.p., Cisplatin 1.5 mg/kg i.p.<br>• Three cycles + 63-day recovery: same BEP schedule, followed by 63 days without chemo, to assess recovery.	• α-Tocopherol 100 mg/kg p.o. once daily<br>• L-ascorbic acid 50 mg/kg p.o. once daily<br>• Selenium 100 µg/L in drinking water		partial recovery after a 63-day recovery period, but did not fully restore normal testicular function or testosterone levels

Razavi et al., 2019	Reproductive (spermatogenesis, sperm parameters, chromatin condensation, seminiferous tubule structure)	Male Wistar rats (180-250 g);<br>BEP chemotherapy administered i.p. on days 1-5 weekly for 9 weeks (3 full cycles):<br>Bleomycin 0.75 mg/kg,<br>Etoposide 7.5 mg/kg,<br>Cisplatin 1.5 mg/kg;<br>Toxicity outcomes assessed at week 18	$ZnSO_4 \cdot 7H_2O$, 10 mg/kg/day orally, for 9 weeks after BEP	• Control (vehicle)<br>• Positive controls (BEP-only)<br>• BEP + then Zinc (post-chemotherapy Zinc)<br>• Zinc only<br>(n = 10/group)	• BEP caused testicular toxicity, including ↓ sperm concentration and motility, ↑ abnormal morphology, poor chromatin condensation (↑ excessive histones), thickened basement membrane, tubular disorganization, ↓ spermatogonia and Leydig cell counts<br>• Zinc after BEP partially reversed toxicity, with significant improvement in sperm concentration, motility, and morphology, improved chromatin condensation, and partial restoration of seminiferous tubule architecture, recovery of spermatogonia and Leydig cells, but not a full return to control levels<br>• Zinc alone showed no adverse effects.
Khadivi et al., 2019	Reproductive (testis and sperm – chromatin integrity and testicular histomorphometry)	Adult male Wistar rats, cancer-free;<br>BEP for 9 weeks:<br>Etoposide 7.5 mg/kg,<br>CIS 1.5 mg/kg i.p. on days 1-5 weekly,<br>Bleomycin 0.75 mg/kg i.p. on day 2 weekly)	$ZnSO_4 \cdot 7H_2O$, 10 mg/kg/day, p.o.,<br>given after the completion of BEP for 9 weeks (approx. one spermatogenesis cycle);<br>Zinc-only group received the same zinc schedule without BEP	• Sham – saline (handling/environ. stress) for 18 weeks<br>• Positive controls (BEP: 9 weeks saline, then 9 weeks BEP)<br>• BEP + Zn: 9 weeks BEP, then 9 weeks zinc<br>• Zinc-only: 9 weeks saline, then 9 weeks zinc<br>(n = 10/group)	• BEP alone caused marked testicular and sperm damage, with ↓global sperm DNA methylation and protamination, ↑DNA fragmentation and ubiquitination of sperm, and degenerative testicular changes (tubular disorganization/atrophy, reduced germinal epithelium thickness, fewer spermatogonia, spermatocytes, and Leydig cells)<br>• Zinc after BEP restored DNA methylation and improved protamination, significantly reduced sperm DNA fragmentation and ubiquitination vs BEP alone, and improved testicular architecture and cell counts (spermatogonia and Leydig cells back to

					control range; partial recovery of spermatocytes and germinal epithelium thickness)<br>• Zinc alone did not impair fertility-related parameters and even increased some germ cell counts vs control
